# Global trends in the awareness of sepsis: insights from search engine data between 2012 and 2017

**DOI:** 10.1186/s13054-017-1914-8

**Published:** 2018-01-17

**Authors:** Craig S. Jabaley, James M. Blum, Robert F. Groff, Vikas N. O’Reilly-Shah

**Affiliations:** 1 Department of Anesthesiology, Division of Critical Care Medicine , Emory University, 1364 Clifton Road NE, Atlanta, GA 30322 USA; 2 Anesthesiology Service Line, Division of Critical Care Medicine, Atlanta Veterans Affairs Medical Center, Decatur, GA USA; 3 Department of Biomedical Informatics, Emory University School of Medicine, Atlanta, GA USA; 4 Department of Anesthesiology, Children’s Healthcare of Atlanta, Atlanta, GA USA

**Keywords:** Sepsis, Disease awareness, Public health, Global health, Autoregressive integrated moving average, Time series analysis

## Abstract

**Background:**

Sepsis is an established global health priority with high mortality that can be curtailed through early recognition and intervention; as such, efforts to raise awareness are potentially impactful and increasingly common. We sought to characterize trends in the awareness of sepsis by examining temporal, geographic, and other changes in search engine utilization for sepsis information-seeking online.

**Methods:**

Using time series analyses and mixed descriptive methods, we retrospectively analyzed publicly available global usage data reported by Google Trends (Google, Palo Alto, CA, USA) concerning web searches for the topic of sepsis between 24 June 2012 and 24 June 2017. Google Trends reports aggregated and de-identified usage data for its search products, including interest over time, interest by region, and details concerning the popularity of related queries where applicable. Outlying epochs of search activity were identified using autoregressive integrated moving average modeling with transfer functions. We then identified awareness campaigns and news media coverage that correlated with epochs of significantly heightened search activity.

**Results:**

A second-order autoregressive model with transfer functions was specified following preliminary outlier analysis. Nineteen significant outlying epochs above the modeled baseline were identified in the final analysis that correlated with 14 awareness and news media events. Our model demonstrated that the baseline level of search activity increased in a nonlinear fashion. A recurrent cyclic increase in search volume beginning in 2012 was observed that correlates with World Sepsis Day. Numerous other awareness and media events were correlated with outlying epochs. The average worldwide search volume for sepsis was less than that of influenza, myocardial infarction, and stroke.

**Conclusions:**

Analyzing aggregate search engine utilization data has promise as a mechanism to measure the impact of awareness efforts. Heightened information-seeking about sepsis occurs in close proximity to awareness events and relevant news media coverage. Future work should focus on validating this approach in other contexts and comparing its results to traditional methods of awareness campaign evaluation.

**Electronic supplementary material:**

The online version of this article (10.1186/s13054-017-1914-8) contains supplementary material, which is available to authorized users.

## Background

Sepsis is a pathophysiologic immune response to infection resulting in potentially fatal organ dysfunction [1–3]. Its high mortality and financial impact have been well described, and early recognition is critical as timely intervention reduces mortality [4–9]. Following the Barcelona Declaration in 2002, campaigns were developed to improve the awareness, recognition, and treatment of sepsis within the medical community [10, 11]. Diagnostic criteria have also been altered to facilitate more rapid recognition [12]. Although evidence suggests that mortality from sepsis in high-income countries is decreasing, its global incidence is increasing, and management challenges in low and middle-income countries (LMIC) confer a disproportionate disease burden [13–19]. Estimates from meta-analyses suggest an annual incidence of 31 million sepsis cases with 6 million deaths; however, the true values are likely even greater [20]. Accordingly, the World Health Organization has adopted a resolution naming sepsis as an important global health priority [21, 22].

Despite its significant global impact, public awareness of sepsis has been historically poor such that 88% of respondents to an international survey in 2009 had never heard the word “sepsis” [23]. In response, major awareness campaigns are being undertaken across the world by both governmental and nongovernmental organizations. The Global Sepsis Alliance, in collaboration with its numerous founding, member, and partner organizations, organized the first annual World Sepsis Day on 13 September 2012 [24, 25]. The US Centers for Disease Control (CDC), the UK National Health Service (NHS), and other such agencies are broadening their awareness efforts beyond the medical community. Simultaneously, high-profile deaths from sepsis have garnered heightened attention from the lay media, and efforts to promote investigational findings through online and traditional media outlets are now commonplace [26–28].

Disease awareness campaigns have grown in popularity, but assessing their impact presents numerous challenges [29, 30]. Surveys tend to be costly, limited in geographic scope, and untimely. Therefore, interest has grown in approaches to understand how healthcare information is disseminated online, and how the public interacts with this information [31]. In the context of public awareness, online information-seeking behaviors can potentially be used as a surrogate measure of disease awareness, because search engines are now routinely leveraged to locate healthcare-related information, including that concerning sepsis [32, 33]. In 2015, one in 20 Google searches was for health-related topics, and common diseases have been incorporated into Google’s hierarchical data structure, known as the Knowledge Graph [34]. Trends in Google search volume have been leveraged to better understand numerous public health issues [35]. We sought to characterize worldwide temporal, geographic, and descriptive trends in online sepsis information-seeking via Google using publicly available usage data to gain further insight into the awareness of sepsis on a large scale. We hypothesized that periods of heightened activity would correlate with awareness events or media coverage related to sepsis.

## Methods

We conducted a retrospective analysis of publicly available data from Google Trends (GT; Google, Mountain View, CA, USA) to explore online sepsis information-seeking behaviors worldwide between 24 June 2012 and 24 June 2017 [36]. An exemption from review was granted by the Emory University Institutional Review Board. Reporting follows the Strengthening the Reporting of Observational Studies in Epidemiology (STROBE) statement guidelines where applicable and those suggested previously by Nuti et al. for reporting of GT search query approaches [35, 37].

### Google Trends methodology

GT reports metrics linked to search queries, including temporal changes in search volume (i.e., interest over time), search volume by geographic region, and related queries. GT returns two categories of related queries on a per-annum basis: top queries, which were the most popular; and rising queries that demonstrated the largest percentage increase in search volume from the prior year. Results and comparisons can be filtered geographically, temporally, categorically, and by type of search. GT characterizes interest over time as the relative search volume (RSV), wherein the peak epoch within the queried timeframe is reported as RSV = 100. GT reports the RSV for either daily or weekly epochs depending on the duration of the queried timeframe, and weekly data were returned for this investigation. We queried GT for web searches with “Sepsis” as a topic (i.e., Freebase machine-generated identifier [MID] /m/014w_8), worldwide, from 24 June 2012 to 24 June 2017, and within all categories. The period of interest was chosen to include the inaugural World Sepsis Day, to exclude the online ahead-of-print publication of a high-visibility editorial concerning the WHO Resolution, and to ensure complete periodicity of the data as discussed subsequently [21]. Querying sepsis as a topic (rather than a search term) leverages a hierarchical data structure and broadly nets relevant related queries, including those in multiple languages. Further methodological considerations concerning GT are outlined in Additional file 1: eMethods 1. The resulting univariate sepsis RSV time series (TS) was exported for further analysis. To better characterize search trends, we exported data regarding interest by region and worldwide top and rising related queries as reported by GT on a per-annum basis during the period of interest.

To ascertain the utilization of Google for healthcare information-seeking and determine the relative frequency of sepsis searches globally, data were extracted from GT on a per-country basis to compare the mean RSV for the topic of sepsis versus the topic of malaria (MID /m/0542n) from 24 June 2007 to 23 June 2012 and then from 24 June 2012 to 24 June 2017. Malaria was chosen as a comparator owing to its importance as a global health issue, which we suspected would portend widespread online information-seeking. A comparative disease multivariate RSV TS was exported to ascertain the popularity over time of sepsis topic web searches versus those for the topics of influenza (MID /m/0cycc), myocardial infarction (MID /m/0gk4g), and stroke (MID /m/02y0js) worldwide during the dates of interest. A US sepsis RSV TS was also exported comprising all web searches for sepsis as a topic within the USA across all categories between 1 January 2012 and 31 December 2016 to facilitate a comparison to available survey data. All final GT queries used for analysis were conducted on 22 July 2017.

### Time series analyses

TS analyses were conducted with R version 3.4.1 (R Core Team, Vienna, Austria) in RStudio 1.0.143 (RStudio, Inc., Boston, MA, USA) and Autobox Enterprise + 6.0.47 (Automatic Forecasting Systems Inc., Hatboro, PA, USA). Detailed technical aspects of all TS analyses are outlined in Additional file 1: eMethods 2. As many TS analytic approaches require a fixed periodicity, the 53rd week in 2015 within the sepsis TS was truncated because forecasting was not a priority. Thereafter the sepsis TS had a weekly periodicity (*N* = 52) over 5 years beginning at the 26th week of 2012 and ending at the 25th week of 2017 with a total of 260 observations. Preliminary classical structural modeling to assess seasonality and the baseline trend was conducted following outlier replacement via linear interpolation, after which a third-order simple moving average (SMA) of the TS was then subjected to additive decomposition by Loess.

Owing to the importance of outliers to the research question, the sepsis TS was modeled using both R and Autobox. For preliminary outlier analysis in R, an autoregressive integrated moving average (ARIMA) model was fit, which is specified by the three parameters *p*, *d*, and *q* referring to the auto-regressive, integrated (i.e., differencing), and moving average orders [38, 39]. ARIMA modeling is a form of regression analysis that examines univariate TS data for the purposes of forecasting and/or identification of outlying values against a specified model. An ARIMA (2,1,2) model was selected via an automated procedure on the basis of the Bayesian information criterion, which penalizes overspecified models [40]. Preliminary outlier detection was accomplished with an adaptation of the procedure described by Chen and Liu, wherein suspected outliers are identified against a specified model with iterative refitting and adjustment to remove those that are nonsignificant [41]. Autobox was used for final modeling with a Box–Jenkins methodology incorporating transfer functions and outlier detection [42, 43]. Suspected causals were specified either a priori or as a result of preliminary outlier detection, thus serving as exogenous predictor variables for dynamic regression. Significance of transfer function coefficients was determined by cross-correlation functions of the prewhitened series.

The comparative disease TS was plotted to facilitate visual comparisons. The US sepsis TS was similarly adjusted for outliers, and a linear regression model was developed to facilitate gross comparisons with longitudinal survey data.

### Descriptive and comparative approach

Outlying values in the sepsis TS were hypothesized to correlate with real world events. Relevant media coverage during the outlying periods was identified through a broad query of Google News limited to the outlying week. Data for related queries were translated using Google Translate where necessary. Per-country mean RSV values for sepsis and malaria from the period of interest and 5 years prior were expressed as a ratio of sepsis:malaria and plotted on choropleths.

## Results

**Fig. 1:**
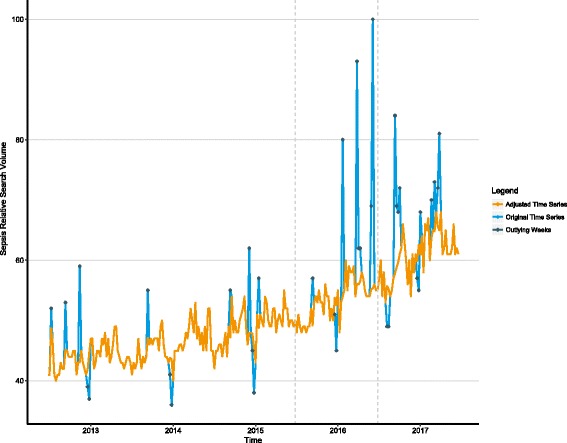
Worldwide relative search volume of queries related to sepsis. Hashed vertical lines represent points at which local time trends were found to change. Outlying weeks and the adjusted time series were generated from the final autoregressive model incorporating transfer functions. Outlying values in the adjusted series have been replaced with those predicted by the final model to highlight the extent of deviation from the modeled baseline. Sepsis was queried as a topic as described in the Methods

Relative to all search queries within a country, queries for the topic of sepsis were the most frequent in Ghana, the UK, Kenya, Ireland, and the USA, respectively (Additional file 1: Table S1). The worldwide sepsis TS was heterogeneous with obvious outlying values and seasonal pulses (Fig. 1, Additional file 1: Table S2). The final model was a second-order autoregressive process plus a constant with 27 transfer functions (root mean squared error = 2.37, *R*^2^ = 0.939; see Additional file 1: eMethods 2). There was no evident overarching seasonal pattern; however, three recurrent seasonal pulses were identified at weeks 37, 51, and 52 beginning in 2012. Exploration of awareness events and news media coverage during each outlying weekly epoch revealed one or more potential correlates (Table 1). Two breakpoints in the baseline trend were detected with local time trends of 0.050 at the beginning of the sepsis TS (24 June 2012), 0.077 starting at 21 June 2015, and 0.024 starting at 26 June 2016. Classical structural modeling likewise suggests that sepsis RSV is increasing in a nonlinear fashion (Additional file 1: Figure S1).

**Table 1:** Individual outlying epochs with relative search volume above the modeled baseline

Week	RSV	Effect duration in weeks (impact per week)	Potential awareness and media correlates	*p* value (per week)
Individual positive outlying epochs				
8 July 2012	52	1 (+3.25)	Pediatric sepsis death in the USA	< 0.0001
11 November 2012	59	1 (+15.95)	Adult death following septic miscarriage in Ireland	< 0.0001
30 November 2014	62	1 (+14.05)	Adult deaths from sepsis secondary to contaminated sharps in India	< 0.0001
11 January 2015	57	1 (+8.26)	Adult sepsis death in the USA	0.0002
24 January 2016	80	1 (+26.0)	NHS report on, and wide coverage of, a pediatric sepsis death in the UK	< 0.0001
27 March 2016	93	3 (+36.86, +6.04, +5.53)	Death of Patty Duke from abdominal sepsis in the USA	< 0.0001, 0.0068, 0.0135
29 May 2016	69	2 (+13.83, +44.66)	Death of Muhammad Ali	< 0.0001, < 0.0001
11 September 2016	84	3 (+18.36, +10.16, +8.33)	Inaugural World Sepsis Congress, fifth World Sepsis Day with international coverage, US CDC awareness efforts	< 0.0001, < 0.0001, 0.0004
2 October 2016	72	1 (+10.95)	Adult sepsis death in the UK	< 0.0001
1 January 2017	68	1 (+7.0)	Pediatric sepsis death in the USA	0.0012
19 February 2017	70	1 (+6.24)	Multiple news media correlates	0.0049
5 March 2017	73	1 (+8.18)	NHS sepsis 1-hour intervention mandate in the UK	0.0003
19 March 2017	72	1 (+6.41)	Coverage of vitamin C as a potential therapeutic intervention	0.0042
26 March 2017	81	1 (+16.03)	Additional vitamin C coverage, NHS apology regarding pediatric sepsis death	< 0.0001
Starting year	Week	Impact coefficient	Potential correlates	
Cyclic outlying effects				
2012	37	+7.82	World Sepsis Day	< 0.0001
2012	51	–2.82	Holiday season	0.0042
2012	52	–7.60		< 0.0001

*CDC* Centers for Disease Control, *NHS* National Health Service, *RSV* relative search volume

**Fig. 2:**
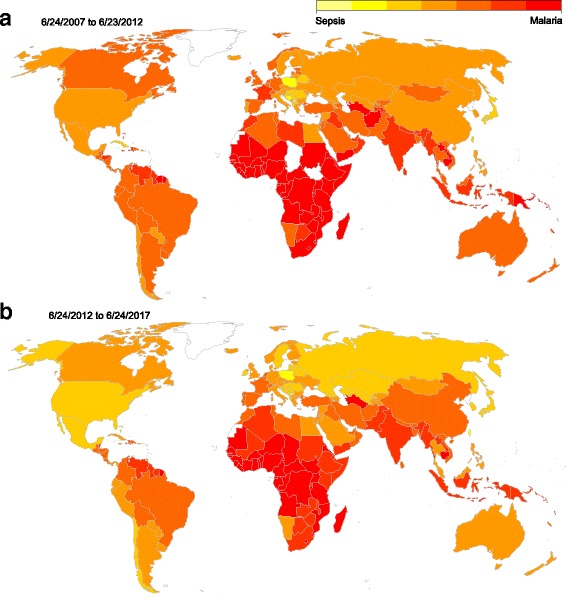
Relative search volume of queries related to sepsis versus malaria. **a** Findings from the 5 years prior to the study period. **b** Findings from the study period. Average relative search volume for sepsis versus malaria is depicted as a ratio on a per-country basis. Sepsis and malaria were queried as topics as described in the Methods

**Fig. 3:**
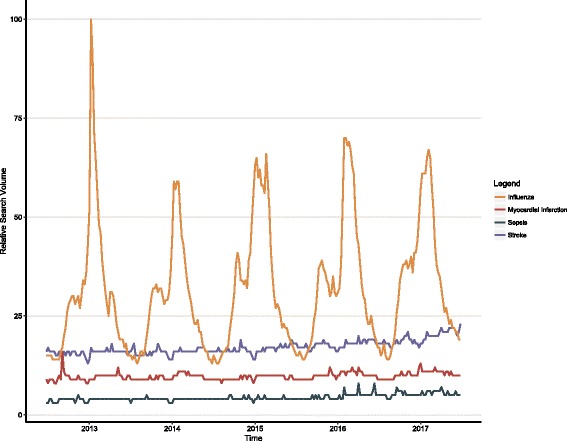
Worldwide relative search volume of queries related to influenza, myocardial infarction, sepsis, and stroke. The comparison is offered to frame the popularity of sepsis searches against those for other common diseases

Top queries related to the topic of sepsis as reported by GT were relatively consistent on a yearly basis and most often represented the words sepsis, septic, or septicemia in one of nine languages (Additional file 1: Table S3). Rising queries as reported by GT were heterogeneous, were germane to the topic of sepsis, and matched correlated events associated with outlying weeks (selected findings in Table 2, full findings in Additional file 1: Table S4). GT returned data regarding queries related to sepsis from 173 countries during the period of interest compared to 169 in the preceding 5 years, and the average RSV for sepsis compared to malaria was 6.7% higher during the period of interest relative to the preceding 5 years (Fig. 2, Additional file 1: Table S5). The average sepsis RSV worldwide during the dates of interest was 4.29, compared to 31.7 for influenza, 17.1 for stroke, and 9.80 for myocardial infarction (Fig. 3, Additional file 1: Table S6). The influenza TS demonstrated marked seasonality, and monthly trends in RSV for stroke and myocardial infarction mirrored the reduction in RSV seen in the sepsis TS at the end of the year. The US sepsis TS linear model had a positive trend of 0.048 per weekly epoch, or an average increase in RSV of 2.5 per year (Additional file 1: Table S7, Figure S2).

**Table 2:** Selected findings from rising related search queries

Themes in rising related search queries	Findings
Languages represented across all reported queries	Chinese, Dutch, French, German, Greek, Indonesian, Italian, Japanese, Korean, Norwegian, Persian, Polish, Portuguese, Russian, Spanish, Thai, Turkish, Ukrainian
Languages represented for “what is sepsis” or “what is septicemia”	English, German, Japanese, Korean, Polish, Portuguese, Russian, Turkish
Languages represented for queries related pediatric sepsis	English, Polish
Queries associated with 2012 guidelines (change from prior year)	surviving sepsis campaign 2012 (140%), surviving sepsis guidelines 2012 (140%), surviving sepsis 2012 (140%), sepsis 2012 (60%)
Queries associated with 2016 guidelines and new diagnostic criteria (change from prior year)	surviving sepsis 2016 (≥ 5000%), sepsis guidelines 2016 (≥ 5000%), jama sepsis 2016 (≥ 5000%), surviving sepsis guidelines 2016 (≥ 5000%), sepsis-3 (3600%), qsofa (3450%), qsofa sepsis (2850%), sepsis 2016 (2050%), sepsis guideline 2016 (2000%), sepsis criteria 2016 (2000%), sofa sepsis (1300%), jama sepsis (1150%), sofa (1100%), new sepsis definition (750%), sofa score sepsis (750%)
Celebrity and high-profile deaths (year in results)	Caden Beggan (2012), Hwang Soo-kwan (2012), Savita Halappanavar (2012), Park Yong-sik (2013), Andressa Urach (2014), Casey Kasem (2014), Kazuo Kawakami (2015), Muhammad Ali (2016), Patty Duke (2016), Guillermo Sanchez (2017)
Awareness events (year in results, change from prior year)	world sepsis day (2012, 650%)
Vitamin C queries in 2017 (change from prior year)	sepsis vitamin c (2150%), vitamin c for sepsis (950%), sepsis cure (100%)
“What is sepsis” per reported language in 2017 (change from 2016)	Japanese (4500%), English (300%), Korean (70%), German (70%)

## Discussion

Online information-seeking about sepsis via Google occurred globally and increased during the studied 5-year timeframe. Awareness and news media correlates were identified for all outlying epochs. The highest RSV values were associated with celebrity deaths, and such deaths accounted for two of the four events with modeled impacts exceeding 1 week. Celebrity names also featured prominently amongst so-called “rising queries” every year. The weeks corresponding to the fifth World Sepsis Day and lay media coverage of vitamin C as a potential therapeutic intervention were the two other events correlated with prolonged effects. The week corresponding to the annual World Sepsis Day on 13 September had a cyclic recurring impact on the modeled baseline. Overall, the underlying sepsis RSV trend was found to be nonlinear, with the greatest average increase in search activity starting in June 2015 and extending to July 2016.

Annual US sepsis awareness surveys demonstrated that 40% of respondents had heard of sepsis in 2012, 44% in 2013–2015, and 55% in 2016 [44]. Consistent with these survey data, we found the increase in sepsis RSV within the USA to be 2.5% per year. Survey respondents were more likely to credit news and entertainment media sources (19% and 13%, respectively) for their awareness of sepsis than medical professionals (12%). Congruently, we found that media coverage appears to be a primary driver of sepsis information-seeking online. Regardless, we would encourage physicians to continue raising awareness of sepsis both during the course of routine clinical care and in conjunction with broader efforts [45]. Although awareness of sepsis may be rising, sepsis RSV was less than that of influenza, stroke, and myocardial infarction despite being prevalent and highly fatal.

Based on these insights from related queries, use of Google to locate information about sepsis is likely undertaken by both the public and healthcare professionals. Speaking to professional interest in sepsis, the findings from 2016 captured searches for updated treatment guidelines and diagnostic criteria coincident with the release of the 2016 Surviving Sepsis Campaign guidelines and Sepsis-3 definitions [11, 12]. It is unlikely that the public would search for “sepsis-3” or “sepsis ICD 10.” Conversely, natural language queries (e.g., “what is sepsis”) are more likely to represent those from the public. As an example, we found that a high-profile death from sepsis in Korea in 2012 was associated with a 550% increase in searches for “what is sepsis” in Korean from the previous year.

Our investigation has several limitations. We examined data from Google as it is the only search engine to make this type of aggregate longitudinal usage data publicly available. Although our findings about searches for malaria suggest that some degree of disease-related information-seeking via Google occurs worldwide, such utilization is likely influenced by accessibility and user preferences. These could be functions of socioeconomic status, geography, politics, or other unknown factors. This may explain why the majority of outlying epochs we identified were correlated with events in the USA and the UK. Another limitation is our inability to definitively identify the cause of increasing search activity. This may reflect increased awareness of sepsis, greater incidence of the disease itself, or other causes we may not be able to enumerate. Indeed, there may be an interplay between such factors. Finally, RSV TS analyses in isolation may not be well suited to capture the impacts of investigational and guideline publications. We identified outlying search activity that correlated with only one such event. However, the impact of these publications became visible on examination of related search query results. Because of the relatively slow diffusion of this knowledge and its impact primarily on only a small proportion of the overall user base (i.e., healthcare professionals), the result of such events may not be evident via RSV TS modeling.

GT has been used previously to both estimate and forecast disease epidemiology with mixed results. Perhaps the most well-known example is that of influenza. The performance of Google Flu was found to be compromised by underling methodological flaws when its estimates were compared against ex-post-facto traditional epidemiological measures. Specifically, fits of the trailing data for forecasting purposes led to systematic overestimates of flu rates [46]. Experience with the now-defunct Google Flu Trends helped to frame our application of GT to questions answerable using this type of data, such as retrospective analyses of the relative interest in various health topics generally, and sepsis specifically.

Data provided from GT must be taken at face value as the underlying methodology for their generation and reporting is dynamic and proprietary. In order to obtain a broad conception of sepsis information-seeking behaviors, we searched using Google Knowledge Graph topics. This approach is broader than a traditional Boolean query but subject to uncertainty as the exact categorization of queries is unknown; however, inferences can be drawn based on the reported top and rising related search queries. For example, “blood infection” emerged as a related term in GT results, and querying the web search interface confirms its linkage to the topic of sepsis as discussed further in Additional file 1: eMethods 1. Not all healthcare topics exist within Google’s hierarchical categorization structure, and thus certain diseases or conditions may not be readily assayable via GT. Google reports search activity as the RSV rather than the raw search volume. This may serve to obfuscate smaller fluctuations in search activity when the queried timeframe includes markedly heightened RSV epochs. Via the AdWords Keyword Planner, Google reported at the time of writing that there are somewhere between 100,000 and 1,000,000 average monthly searches for sepsis both in the USA and the UK, suggesting that the raw search volume represented as the RSV is significant [47].

Furthermore, Google frequently adjusts its algorithmic and reporting methodologies. We encountered a suspected algorithmic change when comparing results from preliminary data-gathering on 6 July 2017 to those obtained from final GT queries on 22 July 2017. The impact thereof was significant as GT later reported an increased sepsis RSV during the 2 weeks correlated with Muhammad Ali’s death (see Additional file 1: eMethods 1 for further discussion). These considerations may impair the ability of other investigators to reproduce our findings by directly querying Google, which is an established limitation [35]. As such, we have made all raw data used in our analysis available in Additional file 1: Tables S1 -- S7. One relatively subtle observation is that there are differences in data availability depending on the query strategy. Querying the average RSV on a per-country basis yielded more granular data from a greater number of individual geographic regions than was reported by the GT interface for a broad worldwide query. Future investigations can leverage this and similar adaptations to improve data resolution.

Our analytic approach likewise has several limitations. First, TS analysis is complex and likely unfamiliar as its primary objective has historically been econometric forecasting. Common approaches involve either expert guidance or the use of costly dedicated software solutions, thus representing a potential barrier for biomedical investigators. However, our findings with both open source and commercial software solutions were similar. Secondly, frequent lay media coverage of sepsis, increasingly visible awareness campaigns, and other such events are all potential factors that could prompt information-seeking behaviors and thus influence the sepsis RSV TS. As such, a quasi-experimental design using either an interrupted TS or a Bayesian causal impact approach was inadvisable. Our approach and final model yielded plausible results in that were able to identify news media and awareness correlates for all outlying RSV epochs. However, causation cannot be established definitively for the association between the posited events and the outlying epochs. Correlated events could likewise be misattributed, and alternative models could conceivably identify different outliers, especially those with subtle effects.

## Conclusions

Examination of search engine utilization trends may yield insights into the success of awareness campaigns. We found that information-seeking behaviors related to sepsis were contemporaneous with awareness campaigns and high-profile media events, such as celebrity deaths. These and related approaches should be developed further to supplement traditional measures as efforts continue to improve the early recognition of sepsis through heightened disease awareness. More broadly, the present approach may be a valuable supplement to the established means by which to evaluate the impact of awareness campaigns in general.

## Additional file

Additional file 1: **eMethods 1:** methodological considerations related to GT, **eMethods 2:** technical aspects of the sepsis (topic) RSV TS analyses, **eReferences:** references within the supplementary online content, **Table S1:** sepsis (topic) RSV by geographic region dataset, **Table S2:** sepsis (topic) RSV TS dataset, **Table S3:** sepsis (topic) top related queries dataset, **Table S4:** sepsis (topic) rising related queries dataset, **Table S5:** average per-country sepsis (topic) versus malaria (topic) RSV dataset, **Table S6:** influenza, myocardial infarction, sepsis, and stroke RSV TS dataset, **Table S7:** sepsis (topic) RSV TS dataset for the USA, **Figure S1:** classical decomposition of the sepsis RSV TS, and **Figure S2:** linear model for the US sepsis (topic) RSV TS. (DOCX 917 kb)

## Abbreviations

ARIMA: Autoregressive integrated moving average
CDC: Centers for Disease Control
GT: Google Trends
LMIC: Low and middle-income countries
MID: Machine-generated identifier
NHS: National Health Service
RSV: Relative search volume
SMA: Simple moving average
STROBE: Strengthening the Reporting of Observational Studies in Epidemiology
TS: Time series
UK: United Kingdom
US: United States
