# Supplementary material for: Global trends in the awareness of sepsis: insights from search engine data between 2012 and 2017
Source: Crit Care. 2018 Jan 17;22:7. doi: 10.1186/s13054-017-1914-8 (PMC5772700; doi:10.1186/s13054-017-1914-8)
Supplement: Additional file 1: — eMethods 1: methodological considerations related to GT, eMethods 2: technical aspects of the sepsis (topic) RSV TS analyses, eReferences: references within the supplementary online content, Table S1: sepsis (topic) RSV by geographic region dataset, Table S2: sepsis (topic) RSV TS dataset, Table S3: sepsis (topic) top related queries dataset, Table S4: sepsis (topic) rising related queries dataset, Table S5: average per-country sepsis (topic) versus malaria (topic) RSV dataset, Table S6: influenza, myocardial infarction, sepsis, and stroke RSV TS dataset, Table S7: sepsis (topic) RSV TS dataset for the USA, Figure S1: classical decomposition of the sepsis RSV TS, and Figure S2: linear model for the US sepsis (topic) RSV TS. (DOCX 917 kb) [file 13054_2017_1914_MOESM1_ESM.docx]

**Additional file 1**

Jabaley C, Blum J, Groff R, O’Reilly-Shah V. Global Trends in the Awareness of Sepsis: Insights from Search Engine Data Between 2012 and 2017.

**eMethods 1.** Methodological Considerations Related to Google Trends

**eMethods 2.** Technical Aspects of the Sepsis (Topic) Relative Search Volume Time Series Analyses

**eReferences.** References Within the Supplementary Online Content

**Table S1.** Sepsis (Topic) Relative Search Volume by Geographic Region Dataset

**Table S2.** Sepsis (Topic) Relative Search Volume Time Series Dataset

**Table S3.** Sepsis (Topic) Top Related Queries Dataset

**Table S4.** Sepsis (Topic) Rising Related Queries Dataset

**Table S5.** Average Per-Country Sepsis (Topic) versus Malaria (Topic) Relative Search Volume Dataset

**Table S6.** Influenza, Myocardial Infarction, Sepsis, and Stroke Relative Search Volume Time Series Dataset

**Table S7.** Sepsis (Topic) Relative Search Volume Time Series Dataset for the United States

**Figure S1.** Classical Decomposition of the Sepsis Relative Search Volume Time Series

**Figure S2.** Linear Model for the United States Sepsis (Topic) Relative Search Volume Time Series

This supplementary material was provided by the authors to give readers additional information about their work.

**eMethods 1. Methodological Considerations Related to Google Trends**

**Background**

Google Trends (GT; Google, Palo Alto, CA) reports aggregate metrics about user interactions with Google search services dating back to 2004.^1^ In the present investigation, GT queries were restricted to web searches, and the discussion herein is limited to that arena. Additional support information was available online from Google at the time of publication.^2^

**Interface**

The primary means by which to interact with GT is through its web interface. An example of its output analogous to that obtained by the authors is provided in eMethods 1, Figure 1. Users first input a search string. At present, GT will accept basic Boolean queries of 15 terms or less. Strings can be queried as either search terms or, where available, as topics within Google’s hierarchical classification (i.e. the Knowledge Graph) as discussed in greater detail subsequently. For example, in eMethods 1, Figure 1, the topic of sepsis (classified as type of injury) maps to the Freebase machine-generated identifier (MID) /m/014w_8. Multiple input strings can be entered using the comparison feature.


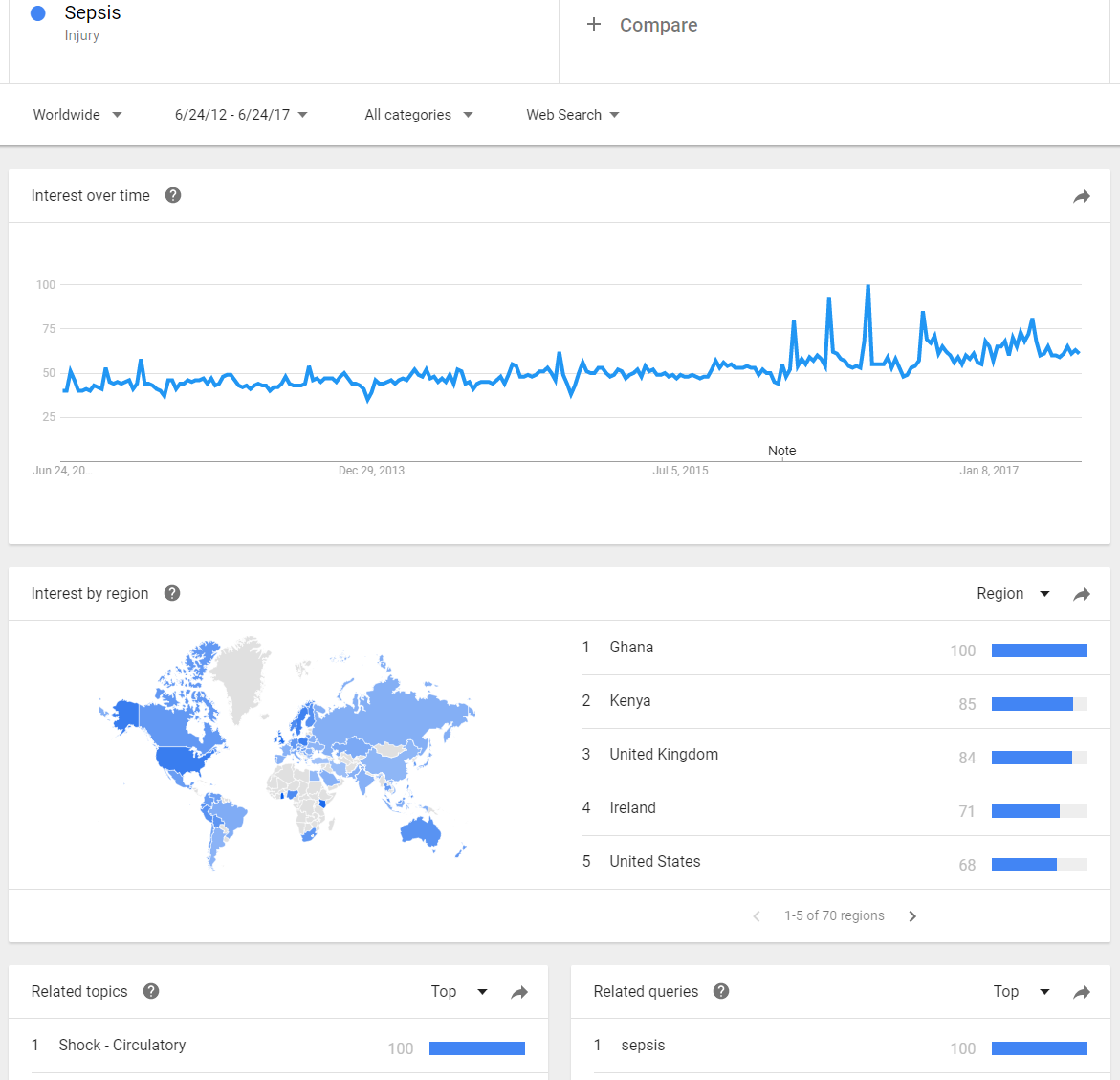


**eMethods 1, Figure 1** – Example Google Trends output for the topic of sepsis. Partial screen capture of <https://trends.google.com/trends/explore?date=2012-06-24%202017-06-24&q=%2Fm%2F014w_8>, accessed 8-15-2017

After specifying a search string within GT, the user is presented with four primary filtering options: location, dates, categories, and search type. Location maps to ISO 3166-1 alpha-2 codes as seen in Table S5. For the present investigation we specified “all categories” and “web search,” although alternative inputs may be appropriate for other investigations.

In response to the above input, GT currently returns four major output categories: interest over time, interest by region, related topics (top and rising), and related queries (top and rising).

Interest over time is depicted as a univariate time series for a single input string or a multivariate time series when using the comparison feature. GT reports interest over time as relative search volume (RSV) wherein the epoch with the highest search volume over the queried timeframe is reported as RSV = 100. GT returns daily values for shorter timeframes and weekly values for longer timeframes. Any dates can be specified; however, for data output on a weekly basis, GT appears to aggregate data with Sunday as the first day of the week. To ensure complete periodicity and capture of relevant data, users may need to specify a timeframe beginning on a Sunday and ending on a Saturday when GT returns weekly values.

Interest by region reports the geographic area in which the queried string represents the greatest proportion of total search activity as RSV = 100. As such, the region with RSV = 100 does not necessarily have the greatest absolute number of searches for the queried string but rather the greatest proportion relative to all search activity. Related topics and related queries are returned under two categories: top and rising. Top results are the most popular with the most commonly searched set as 100. Rising results are those with the greatest increases in popularity compared to the preceding time period, and those with increases of ≥ 5000% are reported as “Breakout.”

All variables can be exported to .csv files using the web interface; however, we observed language encoding issues for non-English characters. These issues can be avoided by copying results directly from the web interface.

**Technical Aspects Concerning Hierarchical Classification**

Specifying strings as a search term returns only results for the string exactly as entered; however, searching by topic nets related queries. For example, inputting sepsis into GT as a search term would return results only for user queries for “sepsis,” whereas searching for sepsis as a topic would return results for queries Google classified as related to the broader concept of sepsis. At present, GT appears to be using Freebase MID codes for topics. Freebase began as an open source project to classify data that was acquired by Google and incorporated into the Knowledge Graph. For topics searched within the accompanying manuscript, the relevant Freebase MID codes utilized by GT are as follows:


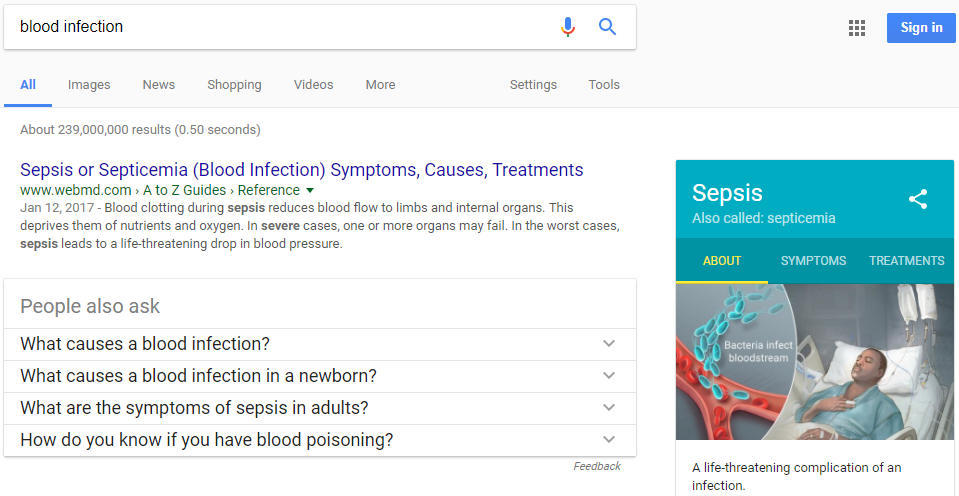


**eMethods 1, Figure 2** – As evidenced by the right-hand pane, Google classifies a query for “blood infection” as being related to the topic of sepsis.
Partial screen capture of <https://www.google.com/search?q=blood+infection>, Accessed 8-22-2017

Sepsis: /m/014w_8
Malaria: /m/0542n
Stroke: /m/02y0js
Myocardial infarction: /m/0gk4g
Influenza: /m/0cycc

Freebase MIDs used by GT are readily apparent in the encoded URL returned by GT. Further exploration of these identifiers can be undertaken through the GT interface, the Knowledge Graph application programming interface (API), or Wikidata .^1,3,4^ At present, the Knowledge Graph API does not return detailed data for many health queries, although Google has undertaken efforts to include health-related queries in their taxonomy.^5,6^ Exploration of the Google web search interface and examination of related results in the output of GT queries can yield insights into Google’s taxonomic classification of healthcare-related queries. For example, a web search for “blood infection” is currently classified as being related to the topic of sepsis as evidenced by Google’s output (eMethods 1, Figure 2). Likewise, searches for sepsis, septicemia, and similar queries in multiple languages currently map to the topic of sepsis. These and similar terms also appeared as related queries during the course of our investigation (Table S3 and S4).

**Additional Means by Which to Access Google Trends Data**

Although GT is not currently accessible via a formal API, automated approaches to data extraction have been developed. We utilized the gtrendsR package for the R environment to facilitate automated data extraction on a per-country basis comparing RSV for sepsis against that for malaria.^7^ In so doing, we noted that data was returned for a larger number of countries compared to a traditional search strategy using the GT GUI. As such, it appears that GT may not report all available data for high-level queries.

**Variability in Data Reported by Google Trends and Algorithmic Changes**

Google’s algorithmic and taxonomic approach to providing search results is dynamic as the underlying methodology is adjusted. This dynamism likewise appears to impact the results of GT queries. We observed variation in the output of GT for an identical query on two different days (7/6/2017 and 7/22/2017) wherein Google later reported greater search volume during the weeks of 5/29/2016 and 6/5/2016 conceivably related to the death of Muhammad Ali from sepsis as suggested by reported top and related queries (eMethods 1, Tables 1 and 2). Variations in the output of GT over time have been noted in other investigations, and changes to GT the reporting schema or the underlying computational or analytic methodology limits reproducibility.^8^ As such, we have reported the raw output of all queries.

**eMethods 1, Table 1** – Google Trends report of top related queries for web searches on the topic of sepsis between 6/24/2012 and 6/24/2017 as reported on two different dates.

| **Results Returned July 6, 2017** | **RSV** | **Results Returned July 22, 2017** | **RSV** |
| --- | --- | --- | --- |
| sepsis | 100 | sepsis | 100 |
| septicemia | 15 | septic | 35 |
| what is sepsis | 5 | septicemia | 15 |
| sepsa | 5 | septic shock | 15 |
| blood infection | 5 | 敗血症 | 5 |
| 敗血症 | 5 | sepsis symptoms | 5 |
| sepsis symptoms | 5 | what is sepsis | 5 |
| septic | 5 | sepse | 5 |
| sepse | 5 | sepsa | 5 |
| blood poisoning | 5 | sepsis infection | 5 |
| sepsis infection | 5 | septic arthritis | 5 |
| сепсис | 5 | neonatal sepsis | 5 |
| sepsis definition | 5 | blood poisoning | 5 |
| septicémie | 5 | сепсис | 5 |
| sepsi | 5 | sepsis definition | 5 |
| sepsis 2016 | 0 | blutvergiftung | 5 |
| blood sepsis | 0 | sepsis shock | 5 |
| setticemia | 0 | septicémie | 5 |
| septicaemia | 0 | septicaemia | 5 |
| symptoms of sepsis | 0 | sepsis 2016 | 0 |
| pneumonia | 0 | sepsi | 0 |
| septic shock | 0 | sepsis icd 10 | 0 |
| sepsis criteria | 0 | what is septic | 0 |
| sepsis signs | 0 | sepsis guidelines | 0 |
| icd 10 sepsis | 0 | setticemia | 0 |

**eMethods 1, Table 2** – Google Trends report of rising related queries for web searches on the topic of sepsis between 6/24/2012 and 6/24/2017 as reported on two different dates

| **Results Returned July 6, 2017** | **ΔRSV** | **Results Returned July 22, 2017** | **ΔRSV** |
| --- | --- | --- | --- |
| patty duke | ≥5000% | patty duke | ≥5000% |
| sepsis ruptured intestine | ≥5000% | muhammad ali | ≥5000% |
| qsofa sepsis | ≥5000% | muhammad ali death | ≥5000% |
| sepsis from a ruptured intestine | ≥5000% | surviving sepsis 2016 | ≥5000% |
| sepsis criteria 2016 | ≥5000% | sepsis guidelines 2016 | ≥5000% |
| sepsis guidelines 2016 | ≥5000% | sepsis from a ruptured intestine | ≥5000% |
| patty duke death | ≥5000% | jama sepsis 2016 | ≥5000% |
| patty duke died | ≥5000% | sepsis ruptured intestine | ≥5000% |
| sepsis-3 | 4650% | surviving sepsis guidelines 2016 | ≥5000% |
| sepses infekcija | 2950% | patty duke death | 4800% |
| nueva definicion de sepsis | 2450% | how did muhammad ali die | 4250% |
| sepsis 2016 | 2300% | patty duke died | 4250% |
| sepsis guideline 2016 | 1750% | sepsis-3 | 3600% |
| sepsis sofa | 1500% | qsofa | 3450% |
| sofa | 1250% | qsofa sepsis | 2850% |
| криптогенный сепсис | 1200% | mohamed ali | 2700% |
| jama sepsis | 1000% | sepsis 2016 | 2050% |
| sofa score sepsis | 900% | sepsis guideline 2016 | 2000% |
| sofa score | 650% | sepsis criteria 2016 | 2000% |
| криптогенный сепсис. | 600% | sofa sepsis | 1300% |
| akut blodförgiftning | 250% | jama sepsis | 1150% |
| new sepsis guidelines | 250% | sofa | 1100% |
| sepsis in babies | 170% | криптогенный сепсис | 1000% |
| icd 10 code sepsis | 160% | new sepsis definition | 750% |
| icd 10 sepsis | 150% | sofa score sepsis | 750% |

**eMethods 2. Technical Aspects of the Sepsis (Topic) Relative Search Volume Time Series Analysis**

**Preliminary Autoregressive Integrated Moving Average (ARIMA) Modeling and Outlier Detection**

Preliminary modeling was conducted in R version 3.4.1 (R Core Team, Vienna, Austria) in RStudio 1.0.143 (RStudio, Inc, Boston, MA, USA using the forecast (Version 8.1), TTR (Version 0.23-2), lmtest (Version 0.9-35), and tsoutliers (Version 0.6-6) packages.^9-13^ Example code and results are provided herein to aid with replication.

require(forecast);

require(TTR);

require(lmtest);

require(tsoutliers);

sepsis_df <- c(41,41,52,46,41,40,41,41,43,42,42,53,45,44,44,44,45,45,41,43,59,45,43,42,41,39,37,47,47,42,43,45,45,44,47,46,48,44,47,43,44,46,49,49,45,44,43,43,42,43,44,44,43,41,43,42,43,47,44,42,44,43,44,55,46,47,46,47,47,47,45,49,50,46,44,44,43,41,36,40,45,45,45,46,46,45,46,48,47,49,52,50,48,53,47,49,46,48,45,49,45,52,52,45,45,42,45,45,46,46,44,46,48,44,49,55,54,48,50,48,48,50,51,52,54,49,46,62,48,45,38,43,51,57,51,50,49,54,53,50,49,49,52,52,48,50,51,50,49,55,52,52,49,50,50,49,50,48,51,49,48,49,49,48,49,49,52,57,53,54,53,55,53,53,51,56,54,54,50,52,50,51,45,44,55,48,53,80,55,60,55,59,58,58,59,54,93,62,62,58,57,55,54,54,54,69,100,56,55,55,57,60,54,58,53,49,49,54,55,57,84,69,68,72,62,66,63,60,56,60,54,61,58,61,57,55,68,64,58,66,65,67,60,70,65,73,68,72,81,68,61,62,65,61,61,61,62,66,61,62,61);

sepsis_df_truncated <- sepsis_df[-184];

sepsis_ts <- ts(sepsis_df_truncated, start=c(2012, 26), freq=52);

tsdisplay(sepsis_ts);

sepsis_ts_clean <- tsclean(sepsis_ts);

fit <- tslm(sepsis_ts_clean~trend);

summary(fit);


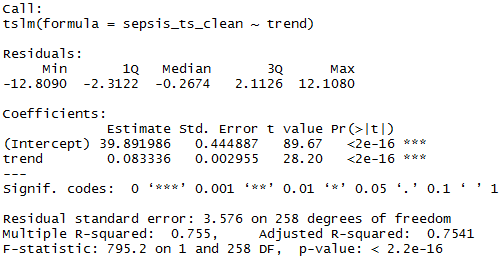


plot(sepsis_ts);

lines(fitted(fit),col="blue");

dwtest(fit);


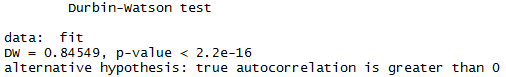


sepsis_ts_SMA3 <- SMA(sepsis_ts_clean, n=3);

sepsis_ts_SMA3_ts <- ts(na.omit(sepsis_ts_SMA3), start=c(2012, 26), freq=52);

sepsis_ts_SMA3_stl <- stl(sepsis_ts_SMA3_ts, "periodic");

plot(sepsis_ts_SMA3_stl);

sepsis_ts_outliers <- tsoutliers::tso(sepsis_ts,types = c("AO","TC","SLS","LS"),maxit.iloop=15,maxit.oloop=30,tsmethod = "auto.arima",args.tsmethod = list(ic = "bic", stepwise = FALSE, stationary = FALSE, seasonal = FALSE, approximation = FALSE));

sepsis_ts_outliers;


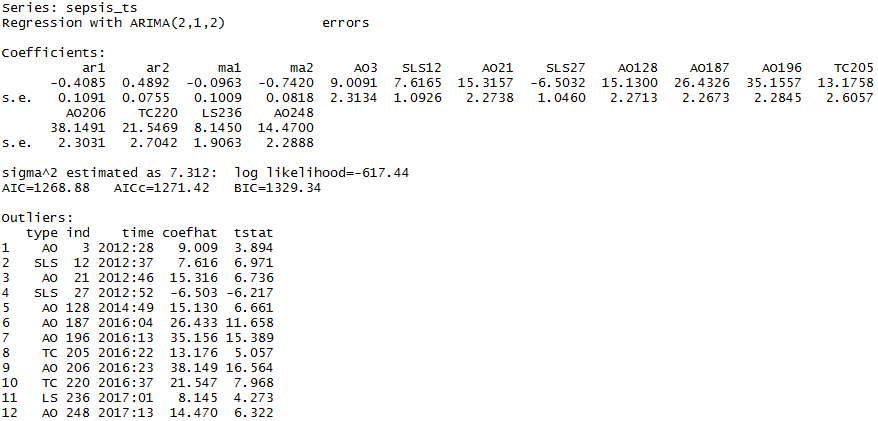


plot(sepsis_ts_outliers);

plot(resid(sepsis_ts_outliers$fit), ylab="Residuals");

Acf(residuals(sepsis_ts_outliers$fit));.

Pacf(residuals(sepsis_ts_outliers$fit));

**Final ARMA Modeling with Transfer Functions**

Following preliminary modeling as above, Autobox Enterprise+ 6.0.47 (Automatic Forecasting Systems INC, Hatboro, PA, USA) was used for final seasonal autoregressive integrated moving average modeling with transfer functions (SARIMAX)

In brief, transfer function modeling seeks to describe the relationship between an output variable (Y) and one, or more, input variables (X). In the case of a time series (Y_1_, Y_2_,…,Y_t_), the impact of input X_t_ on output Y_t_ can be described by the function v(B) (wherein B is a backshift, or lag, operator of the form BX_t_=X_t-1_) plus a constant (c) and a noise (or error) component (N_t_). In a general form, this is expressed as:

$$Y_{t}=c+v\left( B \right)X_{t}+N_{t}$$

v(B) is referred to as a transfer function as it “transfers” changes in X_t_ to Y_t_. The relationship between Y_t_ and X_t_ may not be deterministic owing to noise or an intrinsic dynamic structure; as such ARMA models of orders p and q can proxy stochastic processes expressed as

$$N_{t}=\frac{\theta(B)}{\phi(B)}a_{t}$$

wherein θ describes the MA component of order q, ϕ describes the AR component of order p, and a_t_ is a Gaussian sequence of independent and identically distributed random variables. Similarly, v(B)X_t_ can be expressed as a rational polynomial of varying types.^14^ Thus ARMA modeling with transfer functions for input variables of number i can be summarily expressed as:

$$Y_{t}=c+v\left( B \right)X_{t}+N_{t}=c+\frac{\omega\left( B \right)B^{b}}{\delta\left( B \right)}X_{i,t}+\frac{\theta\left( B \right)}{\phi\left( B \right)}a_{t}$$

wherein b refers to the number of periods over which the effect is delayed, or shifted. Autobox leverages maximum likelihood estimation to conduct time series analysis via SARIMAX modeling, thus applying a partially or fully automated Box-Jenkins approach to time series analysis.^15,16^ Autobox examines model components to ensure that they are necessary (i.e. statistically significant), invertible, and sufficient. A model is fit to the input series and then made stationary by applying said model such that the residuals are reduced to white noise in a process known as prewhitening. Outliers are detected via an approach outlined by Chang and Tiao, implemented by Bell, and further discussed by Tsay.^17-20^ In brief, a series of regressions at each time period is conducted, and in the event of heterogeneous residuals, outliers are represented as intervention variables via transfer functions. Intervention variables can also be specified a priori but will not be included in the final model should they fail to meet checks for necessity and sufficiency. Significance and response weight estimates are assessed via prewhitened cross correlations.

Autobox identified an AR(2) model with 27 transfer functions of the general form

$$Y_{t}=c+X_{1\ldots27,t}+\frac{a_{t}}{1-\phi_{1}B^{1}-\phi_{2}B^{2}}$$

with components as specified in eMethods 2, Table 1. Residual plots are presented in eMethods 2, Figures 1 – 4.

**eMethods 2, Table 1** – Components of the ARMA model with transfer functions as developed with Autobox for the sepsis time series

| Model Component | Coefficient | Period | Year | Week | Type | SE | t-val | p-val |
| --- | --- | --- | --- | --- | --- | --- | --- | --- |
| c | 42.330 |  |  |  | Constant | 3.02 | 7.01 | <0.0001 |
| Φ_1_ | 0.272 |  |  |  | AR Factor 1 | 0.0630 | 4.32 | <0.0001 |
| Φ_2_ | 0.228 |  |  |  | AR Factor 2 | 0.0659 | 3.46 | 0.0007 |
| X12 | + 3.2472 | 3 | 2012 | 28 | Pulse* | 0.704 | 4.62 | <0.0001 |
| X16 | + 15.9491 | 21 | 2012 | 46 | Pulse* | 2.11 | 7.56 | <0.0001 |
| X17 | + 14.0453 | 128 | 2014 | 49 | Pulse*** | 2.12 | 6.62 | <0.0001 |
| X25 | + 8.2616 | 134 | 2015 | 3 | Pulse | 2.15 | 3.85 | 0.0002 |
| X9 | + 25.9996 | 187 | 2016 | 4 | Pulse* | 2.13 | 12.22 | <0.0001 |
| X1 | + 36.8603 | 196 | 2016 | 13 | Pulse* | 2.24 | 16.45 | <0.0001 |
| X10 | + 6.0416 | 197 | 2016 | 14 | Pulse** | 2.21 | 2.73 | 0.0068 |
| X11 | + 5.5309 | 198 | 2016 | 15 | Pulse** | 2.22 | 2.49 | 0.0135 |
| X2 | + 13.8264 | 205 | 2016 | 22 | Pulse* | 2.15 | 6.42 | <0.0001 |
| X4 | + 44.6571 | 206 | 2016 | 23 | Pulse* | 2.15 | 20.73 | <0.0001 |
| X22 | - 6.6886 | 215 | 2016 | 32 | Pulse | 2.16 | -3.09 | 0.0022 |
| X21 | - 6.1932 | 216 | 2016 | 33 | Pulse | 2.15 | -2.87 | 0.0044 |
| X3 | + 18.3608 | 220 | 2016 | 37 | Pulse* | 2.47 | 7.43 | <0.0001 |
| X19 | + 10.1627 | 221 | 2016 | 38 | Pulse* | 2.30 | 4.41 | <0.0001 |
| X23 | + 8.3263 | 222 | 2016 | 39 | Pulse* | 2.30 | 3.62 | 0.0004 |
| X18 | + 10.9494 | 223 | 2016 | 40 | Pulse* | 2.23 | 4.90 | <0.0001 |
| X27 | + 6.9996 | 236 | 2017 | 1 | Pulse*** | 2.13 | 3.28 | 0.0012 |
| X26 | + 6.2402 | 243 | 2017 | 8 | Pulse | 2.20 | 2.84 | 0.0049 |
| X20 | + 8.1817 | 245 | 2017 | 10 | Pulse | 2.21 | 3.70 | 0.0003 |
| X24 | + 6.4147 | 247 | 2017 | 12 | Pulse | 2.22 | 2.89 | 0.0042 |
| X8 | + 16.0324 | 248 | 2017 | 13 | Pulse* | 2.17 | 7.40 | <0.0001 |
| X7 | + 7.8249 | 12 | 2012 | 37 | Seasonal Pulse* | 1.06 | 7.37 | <0.0001 |
| X5 | - 2.8174 | 26 | 2012 | 51 | Seasonal Pulse** | 0.975 | -2.89 | 0.0042 |
| X6 | - 7.6009 | 27 | 2012 | 52 | Seasonal Pulse* | 0.970 | -7.83 | <0.0001 |
| X13 | + 0.0500 | 1 | 2012 | 26 | Time Trend | 0.00736 | 6.80 | <0.0001 |
| X14 | + 0.0766 | 157 | 2015 | 26 | Time Trend | 0.0270 | 2.83 | 0.0050 |
| X15 | + 0.0243 | 209 | 2016 | 26 | Time Trend | 0.0481 | 0.51 | 0.6137 |

Transfer functions are ordered by type and then by period. AR: autoregressive
* Identified in preliminary analysis and manually specified for transfer function modeling
** Not identified in preliminary analysis and manually specified a priori for transfer function modeling
*** Identified in preliminary analysis but not manually specified

**eMethods 2, Figure 1 – Residual plot from the final time series model**


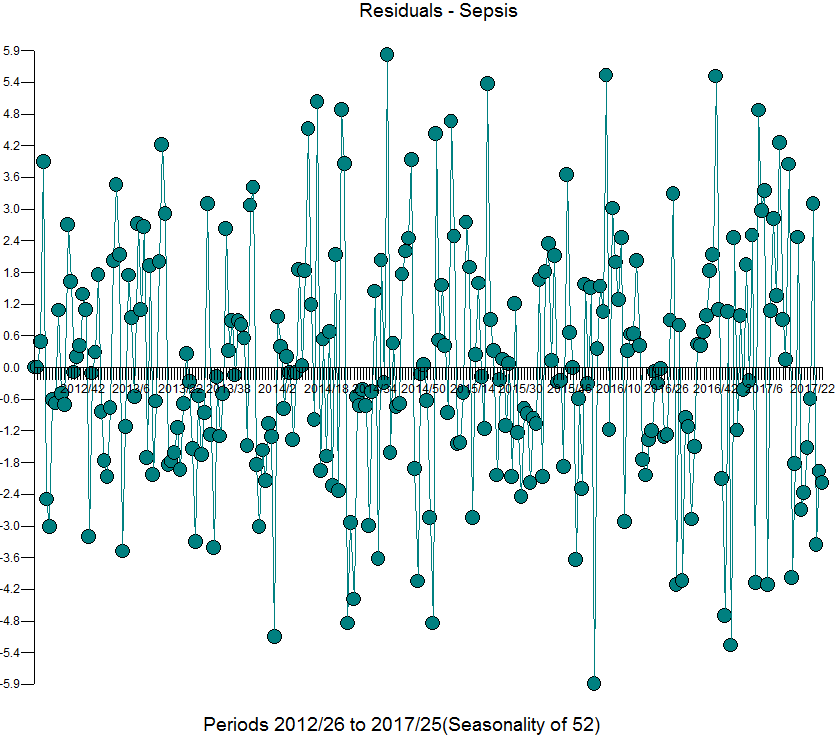


**eMethods 2, Figure 2 – Plot of the autocorrelation function of residuals from the final time series model**


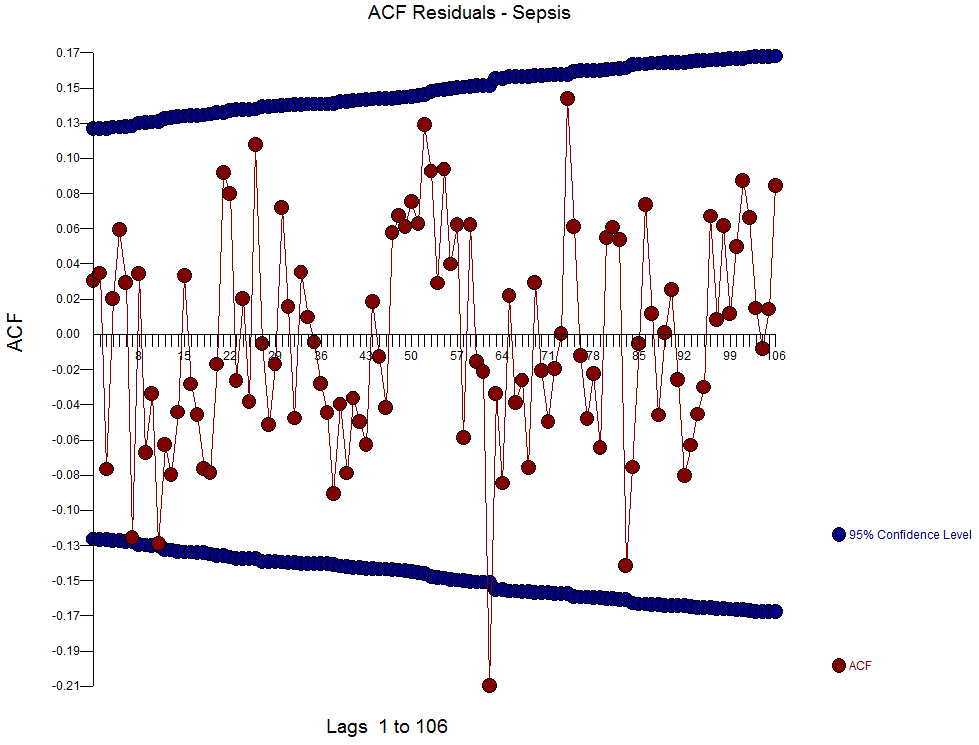


**eMethods 2, Figure 3 – Plot of the partial autocorrelation function of residuals from the final time series model**


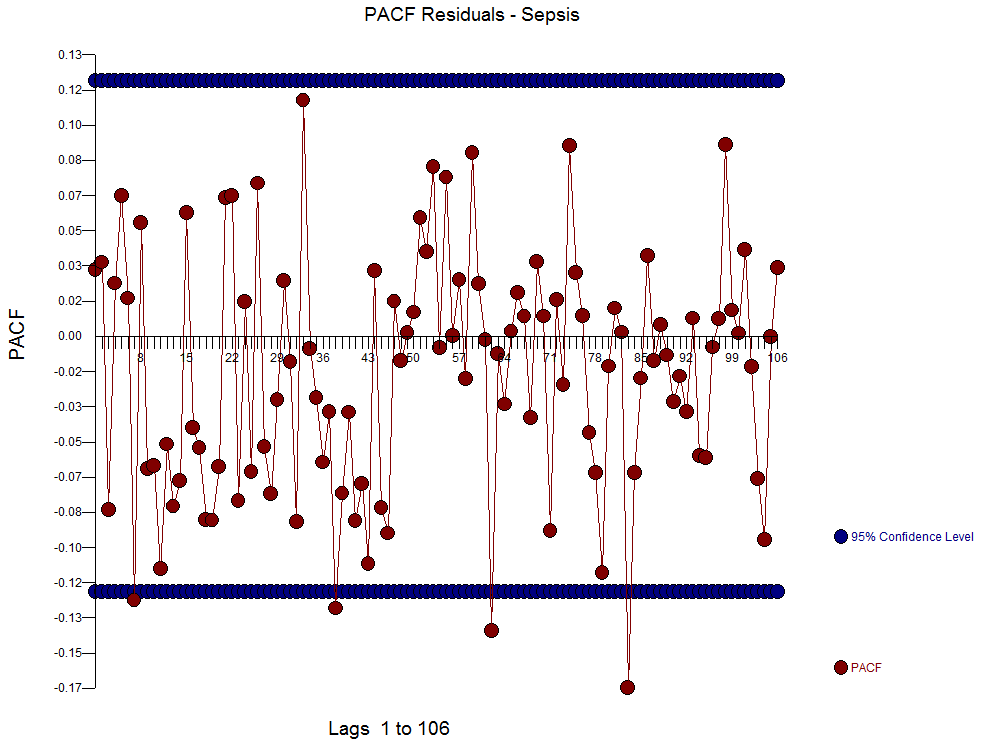


**eMethods 2, Figure 4 – Histogram of residuals from the final time series model**

**eReferences. References Within the Supplementary Online Content**

1. Google Staff. Google Trends. https://trends.google.com/trends/. Accessed July 8, 2017.

2. Google Staff. Google Trends help center. https://support.google.com/trends. Accessed July 8, 2017.

3. Google Staff. Google Knowledge Graph search API. September 3, 2015; https://developers.google.com/knowledge-graph/. Accessed July 8, 2017.

4. Wikidata Contributors. Wikidata. 2017; https://www.wikidata.org.

5. Ramaswami P. A remedy for your health-related questions: health info in the Knowledge Graph. February 10, 2015; https://googleblog.blogspot.com/2015/02/health-info-knowledge-graph.html. Accessed July 8, 2017.

6. Ramaswami P. Now Google can help with updated health information. September 3, 2015; https://search.googleblog.com/2015/09/now-google-can-help-with-updated-health.html. Accessed July 8, 2017.

7. Massicotte P, Eddelbuette D. gtrendsR. 2017; https://github.com/PMassicotte/gtrendsR. Accessed July 22, 2017.

8. Nuti SV, Wayda B, Ranasinghe I, et al. The use of Google Trends in health care research: a systematic review. PLoS One. 2014;9(10):e109583.

9. Hyndman RJ, Khandakar Y. Automatic time series forecasting: the forecast package for R. 2008. 2008;27(3):22.

10. Hyndman RJ. forecast: forecasting functions for time series and linear models. R package version 8.1. 2017; http://github.com/robjhyndman/forecast. Accessed July 8, 2017.

11. Ulrich J. Technical analysis and other functions to construct technical trading rules with R. 2017; https://github.com/joshuaulrich/TTR. Accessed July 8, 2017.

12. Zeileis A, Hothorn T. Diagnostic checking in regression relationships. R News. 2002;2(2):7-10.

13. López-de-Lacalle J. tsoutliers R package for detection of outliers in time series. R package version 0.6-6. 2017; https://jalobe.com/doc/tsoutliers.pdf. Accessed July 8, 2017.

14. Liu L-M, Hanssens DM. Identification of multiple-input transfer function models. Communications in Statistics - Theory and Methods. 1982;11(3):297-314.

15. Box GEP, Jenkins GM. Time series analysis: forecasting and control. San Francisco, CA: Holden-Day; 1976.

16. Reilly D. The AUTOBOX system. Int J Forecasting. 2000;16(4):531-533.

17. Chang I, Tiao GC. Estimation of time series parameters in the presence of outliers. Technical Report #8. Chicago, IL USA: Statistics Research Center, Graduate School of Business, University of Chicago; 1983.

18. Bell WR. A computer program for detecting outliers in time series. Proc Bus Econ Stat Sect. Toronto, Canada: American Statistical Association; 1983:624-639.

19. Tsay RS. Time series model specification in the presence of outliers. J Am Stat Assoc. 1986;81(393):132-141.

20. Tsay RS. Outliers, level shifts, and variance changes in time series. J Forecasting. 1988;7(1):1-20.

**Table S1. Sepsis (Topic) Relative Search Volume by Geographic Region Dataset**

| **Geographic Region** | **Sepsis: (6/24/12 - 6/24/17)** |
| --- | --- |
| Ghana | 100 |
| United Kingdom | 82 |
| Kenya | 80 |
| Ireland | 70 |
| United States | 67 |
| Poland | 65 |
| Sweden | 62 |
| Puerto Rico | 62 |
| Philippines | 59 |
| Nigeria | 56 |
| Norway | 54 |
| Finland | 49 |
| Dominican Republic | 49 |
| Peru | 49 |
| Australia | 49 |
| Ecuador | 49 |
| Bolivia | 47 |
| Malaysia | 46 |
| South Africa | 46 |
| Germany | 45 |
| New Zealand | 44 |
| Canada | 44 |
| Guatemala | 43 |
| South Korea | 43 |
| Switzerland | 43 |
| Taiwan | 42 |
| Thailand | 41 |
| Singapore | 41 |
| Colombia | 41 |
| Croatia | 41 |
| Mexico | 40 |
| Austria | 40 |
| Denmark | 39 |
| Venezuela | 38 |
| Chile | 38 |
| Hungary | 35 |
| Portugal | 34 |
| Lithuania | 33 |
| Czechia | 32 |
| Netherlands | 31 |
| Italy | 31 |
| Kazakhstan | 30 |
| Slovakia | 30 |
| Japan | 29 |
| Hong Kong | 29 |
| Jordan | 29 |
| Serbia | 28 |
| Brazil | 27 |
| Belgium | 27 |
| United Arab Emirates | 27 |
| Indonesia | 26 |
| Belarus | 25 |
| Russia | 25 |
| Pakistan | 25 |
| India | 25 |
| Spain | 24 |
| Saudi Arabia | 24 |
| Greece | 23 |
| France | 23 |
| Romania | 23 |
| Argentina | 21 |
| Ukraine | 21 |
| China | 19 |
| Israel | 19 |
| Vietnam | 18 |
| Egypt | 18 |
| Bulgaria | 16 |
| Iran | 13 |
| Turkey | 10 |

**Table S2. Sepsis (Topic) Relative Search Volume Time Series Dataset**

| **Week Start** | **Sepsis: (6/24/12 - 6/24/17)** | **Period** | **Year** | **Week** |
| --- | --- | --- | --- | --- |
| 6/24/2012 | 41 | 1 | 2012 | 26 |
| 7/1/2012 | 41 | 2 | 2012 | 27 |
| 7/8/2012 | 52 | 3 | 2012 | 28 |
| 7/15/2012 | 46 | 4 | 2012 | 29 |
| 7/22/2012 | 41 | 5 | 2012 | 30 |
| 7/29/2012 | 40 | 6 | 2012 | 31 |
| 8/5/2012 | 41 | 7 | 2012 | 32 |
| 8/12/2012 | 41 | 8 | 2012 | 33 |
| 8/19/2012 | 43 | 9 | 2012 | 34 |
| 8/26/2012 | 42 | 10 | 2012 | 35 |
| 9/2/2012 | 42 | 11 | 2012 | 36 |
| 9/9/2012 | 53 | 12 | 2012 | 37 |
| 9/16/2012 | 45 | 13 | 2012 | 38 |
| 9/23/2012 | 44 | 14 | 2012 | 39 |
| 9/30/2012 | 44 | 15 | 2012 | 40 |
| 10/7/2012 | 44 | 16 | 2012 | 41 |
| 10/14/2012 | 45 | 17 | 2012 | 42 |
| 10/21/2012 | 45 | 18 | 2012 | 43 |
| 10/28/2012 | 41 | 19 | 2012 | 44 |
| 11/4/2012 | 43 | 20 | 2012 | 45 |
| 11/11/2012 | 59 | 21 | 2012 | 46 |
| 11/18/2012 | 45 | 22 | 2012 | 47 |
| 11/25/2012 | 43 | 23 | 2012 | 48 |
| 12/2/2012 | 42 | 24 | 2012 | 49 |
| 12/9/2012 | 41 | 25 | 2012 | 50 |
| 12/16/2012 | 39 | 26 | 2012 | 51 |
| 12/23/2012 | 37 | 27 | 2012 | 52 |
| 12/30/2012 | 47 | 28 | 2013 | 1 |
| 1/6/2013 | 47 | 29 | 2013 | 2 |
| 1/13/2013 | 42 | 30 | 2013 | 3 |
| 1/20/2013 | 43 | 31 | 2013 | 4 |
| 1/27/2013 | 45 | 32 | 2013 | 5 |
| 2/3/2013 | 45 | 33 | 2013 | 6 |
| 2/10/2013 | 44 | 34 | 2013 | 7 |
| 2/17/2013 | 47 | 35 | 2013 | 8 |
| 2/24/2013 | 46 | 36 | 2013 | 9 |
| 3/3/2013 | 48 | 37 | 2013 | 10 |
| 3/10/2013 | 44 | 38 | 2013 | 11 |
| 3/17/2013 | 47 | 39 | 2013 | 12 |
| 3/24/2013 | 43 | 40 | 2013 | 13 |
| 3/31/2013 | 44 | 41 | 2013 | 14 |
| 4/7/2013 | 46 | 42 | 2013 | 15 |
| 4/14/2013 | 49 | 43 | 2013 | 16 |
| 4/21/2013 | 49 | 44 | 2013 | 17 |
| 4/28/2013 | 45 | 45 | 2013 | 18 |
| 5/5/2013 | 44 | 46 | 2013 | 19 |
| 5/12/2013 | 43 | 47 | 2013 | 20 |
| 5/19/2013 | 43 | 48 | 2013 | 21 |
| 5/26/2013 | 42 | 49 | 2013 | 22 |
| 6/2/2013 | 43 | 50 | 2013 | 23 |
| 6/9/2013 | 44 | 51 | 2013 | 24 |
| 6/16/2013 | 44 | 52 | 2013 | 25 |
| 6/23/2013 | 43 | 53 | 2013 | 26 |
| 6/30/2013 | 41 | 54 | 2013 | 27 |
| 7/7/2013 | 43 | 55 | 2013 | 28 |
| 7/14/2013 | 42 | 56 | 2013 | 29 |
| 7/21/2013 | 43 | 57 | 2013 | 30 |
| 7/28/2013 | 47 | 58 | 2013 | 31 |
| 8/4/2013 | 44 | 59 | 2013 | 32 |
| 8/11/2013 | 42 | 60 | 2013 | 33 |
| 8/18/2013 | 44 | 61 | 2013 | 34 |
| 8/25/2013 | 43 | 62 | 2013 | 35 |
| 9/1/2013 | 44 | 63 | 2013 | 36 |
| 9/8/2013 | 55 | 64 | 2013 | 37 |
| 9/15/2013 | 46 | 65 | 2013 | 38 |
| 9/22/2013 | 47 | 66 | 2013 | 39 |
| 9/29/2013 | 46 | 67 | 2013 | 40 |
| 10/6/2013 | 47 | 68 | 2013 | 41 |
| 10/13/2013 | 47 | 69 | 2013 | 42 |
| 10/20/2013 | 47 | 70 | 2013 | 43 |
| 10/27/2013 | 45 | 71 | 2013 | 44 |
| 11/3/2013 | 49 | 72 | 2013 | 45 |
| 11/10/2013 | 50 | 73 | 2013 | 46 |
| 11/17/2013 | 46 | 74 | 2013 | 47 |
| 11/24/2013 | 44 | 75 | 2013 | 48 |
| 12/1/2013 | 44 | 76 | 2013 | 49 |
| 12/8/2013 | 43 | 77 | 2013 | 50 |
| 12/15/2013 | 41 | 78 | 2013 | 51 |
| 12/22/2013 | 36 | 79 | 2013 | 52 |
| 12/29/2013 | 40 | 80 | 2014 | 1 |
| 1/5/2014 | 45 | 81 | 2014 | 2 |
| 1/12/2014 | 45 | 82 | 2014 | 3 |
| 1/19/2014 | 45 | 83 | 2014 | 4 |
| 1/26/2014 | 46 | 84 | 2014 | 5 |
| 2/2/2014 | 46 | 85 | 2014 | 6 |
| 2/9/2014 | 45 | 86 | 2014 | 7 |
| 2/16/2014 | 46 | 87 | 2014 | 8 |
| 2/23/2014 | 48 | 88 | 2014 | 9 |
| 3/2/2014 | 47 | 89 | 2014 | 10 |
| 3/9/2014 | 49 | 90 | 2014 | 11 |
| 3/16/2014 | 52 | 91 | 2014 | 12 |
| 3/23/2014 | 50 | 92 | 2014 | 13 |
| 3/30/2014 | 48 | 93 | 2014 | 14 |
| 4/6/2014 | 53 | 94 | 2014 | 15 |
| 4/13/2014 | 47 | 95 | 2014 | 16 |
| 4/20/2014 | 49 | 96 | 2014 | 17 |
| 4/27/2014 | 46 | 97 | 2014 | 18 |
| 5/4/2014 | 48 | 98 | 2014 | 19 |
| 5/11/2014 | 45 | 99 | 2014 | 20 |
| 5/18/2014 | 49 | 100 | 2014 | 21 |
| 5/25/2014 | 45 | 101 | 2014 | 22 |
| 6/1/2014 | 52 | 102 | 2014 | 23 |
| 6/8/2014 | 52 | 103 | 2014 | 24 |
| 6/15/2014 | 45 | 104 | 2014 | 25 |
| 6/22/2014 | 45 | 105 | 2014 | 26 |
| 6/29/2014 | 42 | 106 | 2014 | 27 |
| 7/6/2014 | 45 | 107 | 2014 | 28 |
| 7/13/2014 | 45 | 108 | 2014 | 29 |
| 7/20/2014 | 46 | 109 | 2014 | 30 |
| 7/27/2014 | 46 | 110 | 2014 | 31 |
| 8/3/2014 | 44 | 111 | 2014 | 32 |
| 8/10/2014 | 46 | 112 | 2014 | 33 |
| 8/17/2014 | 48 | 113 | 2014 | 34 |
| 8/24/2014 | 44 | 114 | 2014 | 35 |
| 8/31/2014 | 49 | 115 | 2014 | 36 |
| 9/7/2014 | 55 | 116 | 2014 | 37 |
| 9/14/2014 | 54 | 117 | 2014 | 38 |
| 9/21/2014 | 48 | 118 | 2014 | 39 |
| 9/28/2014 | 50 | 119 | 2014 | 40 |
| 10/5/2014 | 48 | 120 | 2014 | 41 |
| 10/12/2014 | 48 | 121 | 2014 | 42 |
| 10/19/2014 | 50 | 122 | 2014 | 43 |
| 10/26/2014 | 51 | 123 | 2014 | 44 |
| 11/2/2014 | 52 | 124 | 2014 | 45 |
| 11/9/2014 | 54 | 125 | 2014 | 46 |
| 11/16/2014 | 49 | 126 | 2014 | 47 |
| 11/23/2014 | 46 | 127 | 2014 | 48 |
| 11/30/2014 | 62 | 128 | 2014 | 49 |
| 12/7/2014 | 48 | 129 | 2014 | 50 |
| 12/14/2014 | 45 | 130 | 2014 | 51 |
| 12/21/2014 | 38 | 131 | 2014 | 52 |
| 12/28/2014 | 43 | 132 | 2015 | 1 |
| 1/4/2015 | 51 | 133 | 2015 | 2 |
| 1/11/2015 | 57 | 134 | 2015 | 3 |
| 1/18/2015 | 51 | 135 | 2015 | 4 |
| 1/25/2015 | 50 | 136 | 2015 | 5 |
| 2/1/2015 | 49 | 137 | 2015 | 6 |
| 2/8/2015 | 54 | 138 | 2015 | 7 |
| 2/15/2015 | 53 | 139 | 2015 | 8 |
| 2/22/2015 | 50 | 140 | 2015 | 9 |
| 3/1/2015 | 49 | 141 | 2015 | 10 |
| 3/8/2015 | 49 | 142 | 2015 | 11 |
| 3/15/2015 | 52 | 143 | 2015 | 12 |
| 3/22/2015 | 52 | 144 | 2015 | 13 |
| 3/29/2015 | 48 | 145 | 2015 | 14 |
| 4/5/2015 | 50 | 146 | 2015 | 15 |
| 4/12/2015 | 51 | 147 | 2015 | 16 |
| 4/19/2015 | 50 | 148 | 2015 | 17 |
| 4/26/2015 | 49 | 149 | 2015 | 18 |
| 5/3/2015 | 55 | 150 | 2015 | 19 |
| 5/10/2015 | 52 | 151 | 2015 | 20 |
| 5/17/2015 | 52 | 152 | 2015 | 21 |
| 5/24/2015 | 49 | 153 | 2015 | 22 |
| 5/31/2015 | 50 | 154 | 2015 | 23 |
| 6/7/2015 | 50 | 155 | 2015 | 24 |
| 6/14/2015 | 49 | 156 | 2015 | 25 |
| 6/21/2015 | 50 | 157 | 2015 | 26 |
| 6/28/2015 | 48 | 158 | 2015 | 27 |
| 7/5/2015 | 51 | 159 | 2015 | 28 |
| 7/12/2015 | 49 | 160 | 2015 | 29 |
| 7/19/2015 | 48 | 161 | 2015 | 30 |
| 7/26/2015 | 49 | 162 | 2015 | 31 |
| 8/2/2015 | 49 | 163 | 2015 | 32 |
| 8/9/2015 | 48 | 164 | 2015 | 33 |
| 8/16/2015 | 49 | 165 | 2015 | 34 |
| 8/23/2015 | 49 | 166 | 2015 | 35 |
| 8/30/2015 | 52 | 167 | 2015 | 36 |
| 9/6/2015 | 57 | 168 | 2015 | 37 |
| 9/13/2015 | 53 | 169 | 2015 | 38 |
| 9/20/2015 | 54 | 170 | 2015 | 39 |
| 9/27/2015 | 53 | 171 | 2015 | 40 |
| 10/4/2015 | 55 | 172 | 2015 | 41 |
| 10/11/2015 | 53 | 173 | 2015 | 42 |
| 10/18/2015 | 53 | 174 | 2015 | 43 |
| 10/25/2015 | 51 | 175 | 2015 | 44 |
| 11/1/2015 | 56 | 176 | 2015 | 45 |
| 11/8/2015 | 54 | 177 | 2015 | 46 |
| 11/15/2015 | 54 | 178 | 2015 | 47 |
| 11/22/2015 | 50 | 179 | 2015 | 48 |
| 11/29/2015 | 52 | 180 | 2015 | 49 |
| 12/6/2015 | 50 | 181 | 2015 | 50 |
| 12/13/2015 | 51 | 182 | 2015 | 51 |
| 12/20/2015 | 45 | 183 | 2015 | 52 |
| 12/27/2015 | 44 | 184 | 2015 | 53 |
| 1/3/2016 | 55 | 185 | 2016 | 1 |
| 1/10/2016 | 48 | 186 | 2016 | 2 |
| 1/17/2016 | 53 | 187 | 2016 | 3 |
| 1/24/2016 | 80 | 188 | 2016 | 4 |
| 1/31/2016 | 55 | 189 | 2016 | 5 |
| 2/7/2016 | 60 | 190 | 2016 | 6 |
| 2/14/2016 | 55 | 191 | 2016 | 7 |
| 2/21/2016 | 59 | 192 | 2016 | 8 |
| 2/28/2016 | 58 | 193 | 2016 | 9 |
| 3/6/2016 | 58 | 194 | 2016 | 10 |
| 3/13/2016 | 59 | 195 | 2016 | 11 |
| 3/20/2016 | 54 | 196 | 2016 | 12 |
| 3/27/2016 | 93 | 197 | 2016 | 13 |
| 4/3/2016 | 62 | 198 | 2016 | 14 |
| 4/10/2016 | 62 | 199 | 2016 | 15 |
| 4/17/2016 | 58 | 200 | 2016 | 16 |
| 4/24/2016 | 57 | 201 | 2016 | 17 |
| 5/1/2016 | 55 | 202 | 2016 | 18 |
| 5/8/2016 | 54 | 203 | 2016 | 19 |
| 5/15/2016 | 54 | 204 | 2016 | 20 |
| 5/22/2016 | 54 | 205 | 2016 | 21 |
| 5/29/2016 | 69 | 206 | 2016 | 22 |
| 6/5/2016 | 100 | 207 | 2016 | 23 |
| 6/12/2016 | 56 | 208 | 2016 | 24 |
| 6/19/2016 | 55 | 209 | 2016 | 25 |
| 6/26/2016 | 55 | 210 | 2016 | 26 |
| 7/3/2016 | 57 | 211 | 2016 | 27 |
| 7/10/2016 | 60 | 212 | 2016 | 28 |
| 7/17/2016 | 54 | 213 | 2016 | 29 |
| 7/24/2016 | 58 | 214 | 2016 | 30 |
| 7/31/2016 | 53 | 215 | 2016 | 31 |
| 8/7/2016 | 49 | 216 | 2016 | 32 |
| 8/14/2016 | 49 | 217 | 2016 | 33 |
| 8/21/2016 | 54 | 218 | 2016 | 34 |
| 8/28/2016 | 55 | 219 | 2016 | 35 |
| 9/4/2016 | 57 | 220 | 2016 | 36 |
| 9/11/2016 | 84 | 221 | 2016 | 37 |
| 9/18/2016 | 69 | 222 | 2016 | 38 |
| 9/25/2016 | 68 | 223 | 2016 | 39 |
| 10/2/2016 | 72 | 224 | 2016 | 40 |
| 10/9/2016 | 62 | 225 | 2016 | 41 |
| 10/16/2016 | 66 | 226 | 2016 | 42 |
| 10/23/2016 | 63 | 227 | 2016 | 43 |
| 10/30/2016 | 60 | 228 | 2016 | 44 |
| 11/6/2016 | 56 | 229 | 2016 | 45 |
| 11/13/2016 | 60 | 230 | 2016 | 46 |
| 11/20/2016 | 54 | 231 | 2016 | 47 |
| 11/27/2016 | 61 | 232 | 2016 | 48 |
| 12/4/2016 | 58 | 233 | 2016 | 49 |
| 12/11/2016 | 61 | 234 | 2016 | 50 |
| 12/18/2016 | 57 | 235 | 2016 | 51 |
| 12/25/2016 | 55 | 236 | 2016 | 52 |
| 1/1/2017 | 68 | 237 | 2017 | 1 |
| 1/8/2017 | 64 | 238 | 2017 | 2 |
| 1/15/2017 | 58 | 239 | 2017 | 3 |
| 1/22/2017 | 66 | 240 | 2017 | 4 |
| 1/29/2017 | 65 | 241 | 2017 | 5 |
| 2/5/2017 | 67 | 242 | 2017 | 6 |
| 2/12/2017 | 60 | 243 | 2017 | 7 |
| 2/19/2017 | 70 | 244 | 2017 | 8 |
| 2/26/2017 | 65 | 245 | 2017 | 9 |
| 3/5/2017 | 73 | 246 | 2017 | 10 |
| 3/12/2017 | 68 | 247 | 2017 | 11 |
| 3/19/2017 | 72 | 248 | 2017 | 12 |
| 3/26/2017 | 81 | 249 | 2017 | 13 |
| 4/2/2017 | 68 | 250 | 2017 | 14 |
| 4/9/2017 | 61 | 251 | 2017 | 15 |
| 4/16/2017 | 62 | 252 | 2017 | 16 |
| 4/23/2017 | 65 | 253 | 2017 | 17 |
| 4/30/2017 | 61 | 254 | 2017 | 18 |
| 5/7/2017 | 61 | 255 | 2017 | 19 |
| 5/14/2017 | 61 | 256 | 2017 | 20 |
| 5/21/2017 | 62 | 257 | 2017 | 21 |
| 5/28/2017 | 66 | 258 | 2017 | 22 |
| 6/4/2017 | 61 | 259 | 2017 | 23 |
| 6/11/2017 | 62 | 260 | 2017 | 24 |
| 6/18/2017 | 61 | 261 | 2017 | 25 |

**Table S3. Sepsis (Topic) Top Related Queries Dataset**

| **Year** | **Query** | **RSV** |
| --- | --- | --- |
| 2012 | sepsis | 100 |
| 2012 | septic | 40 |
| 2012 | septicemia | 25 |
| 2012 | shock | 15 |
| 2012 | septic shock | 15 |
| 2012 | 敗血症 | 10 |
| 2012 | sepsa | 10 |
| 2012 | blood poisoning | 5 |
| 2012 | septicaemia | 5 |
| 2012 | blutvergiftung | 5 |
| 2012 | arthritis | 5 |
| 2012 | sepse | 5 |
| 2012 | septic arthritis | 5 |
| 2012 | septicémie | 5 |
| 2012 | sepsis neonatal | 5 |
| 2012 | sepsis symptoms | 5 |
| 2012 | sepsis infection | 5 |
| 2012 | what is sepsis | 5 |
| 2012 | sepsi | 5 |
| 2012 | сепсис | 5 |
| 2012 | sepsis shock | 5 |
| 2012 | sepsis guidelines | 5 |
| 2012 | setticemia | 5 |
| 2012 | septic infection | 5 |
| 2012 | surviving sepsis | 0 |
| 2013 | sepsis | 100 |
| 2013 | septicemia | 25 |
| 2013 | sepsa | 10 |
| 2013 | 敗血症 | 5 |
| 2013 | blood infection | 5 |
| 2013 | septic | 5 |
| 2013 | blood poisoning | 5 |
| 2013 | sepse | 5 |
| 2013 | septicémie | 5 |
| 2013 | sepsis symptoms | 5 |
| 2013 | what is sepsis | 5 |
| 2013 | septicaemia | 5 |
| 2013 | sepsi | 5 |
| 2013 | сепсис | 5 |
| 2013 | sepsis infection | 5 |
| 2013 | sepsis shock | 5 |
| 2013 | setticemia | 5 |
| 2013 | posocznica | 0 |
| 2013 | septic shock | 0 |
| 2013 | sepsis guidelines | 0 |
| 2013 | septic infection | 0 |
| 2013 | sepsis treatment | 0 |
| 2013 | pneumonia | 0 |
| 2013 | definition sepsis | 0 |
| 2013 | septicemie | 0 |
| 2014 | sepsis | 100 |
| 2014 | septicemia | 25 |
| 2014 | sepsa | 10 |
| 2014 | 敗血症 | 10 |
| 2014 | blood infection | 5 |
| 2014 | septic | 5 |
| 2014 | sepse | 5 |
| 2014 | blood poisoning | 5 |
| 2014 | septicémie | 5 |
| 2014 | what is sepsis | 5 |
| 2014 | sepsis symptoms | 5 |
| 2014 | sepsi | 5 |
| 2014 | sepsis infection | 5 |
| 2014 | сепсис | 5 |
| 2014 | septicaemia | 5 |
| 2014 | setticemia | 5 |
| 2014 | sepsis definition | 0 |
| 2014 | septic shock | 0 |
| 2014 | sepsis treatment | 0 |
| 2014 | pneumonia | 0 |
| 2014 | sepsis criteria | 0 |
| 2014 | severe sepsis | 0 |
| 2014 | sepsis guidelines | 0 |
| 2014 | septicemie | 0 |
| 2014 | sirs | 0 |
| 2015 | sepsis | 100 |
| 2015 | septicemia | 20 |
| 2015 | sepsa | 10 |
| 2015 | 敗血症 | 10 |
| 2015 | blood infection | 5 |
| 2015 | septic | 5 |
| 2015 | sepse | 5 |
| 2015 | what is sepsis | 5 |
| 2015 | blood poisoning | 5 |
| 2015 | sepsis symptoms | 5 |
| 2015 | sepsis infection | 5 |
| 2015 | сепсис | 5 |
| 2015 | septicémie | 5 |
| 2015 | sepsi | 5 |
| 2015 | blood sepsis | 5 |
| 2015 | septicaemia | 5 |
| 2015 | sepsis shock | 5 |
| 2015 | setticemia | 0 |
| 2015 | sepsis definition | 0 |
| 2015 | sepsis criteria | 0 |
| 2015 | pneumonia | 0 |
| 2015 | septic shock | 0 |
| 2015 | sirs | 0 |
| 2015 | sepsis treatment | 0 |
| 2015 | severe sepsis | 0 |
| 2016 | sepsis | 100 |
| 2016 | septic | 35 |
| 2016 | septicemia | 15 |
| 2016 | septic shock | 15 |
| 2016 | 敗血症 | 5 |
| 2016 | sepsis symptoms | 5 |
| 2016 | what is sepsis | 5 |
| 2016 | sepse | 5 |
| 2016 | sepsa | 5 |
| 2016 | sepsis infection | 5 |
| 2016 | septic arthritis | 5 |
| 2016 | neonatal sepsis | 5 |
| 2016 | blood poisoning | 5 |
| 2016 | сепсис | 5 |
| 2016 | sepsis definition | 5 |
| 2016 | blutvergiftung | 5 |
| 2016 | sepsis shock | 5 |
| 2016 | septicémie | 5 |
| 2016 | septicaemia | 5 |
| 2016 | sepsis 2016 | 0 |
| 2016 | sepsi | 0 |
| 2016 | sepsis icd 10 | 0 |
| 2016 | what is septic | 0 |
| 2016 | sepsis guidelines | 0 |
| 2016 | setticemia | 0 |
| 2017 | sepsis | 100 |
| 2017 | septicemia | 15 |
| 2017 | infection | 10 |
| 2017 | what is sepsis | 5 |
| 2017 | sepsa | 5 |
| 2017 | 敗血症 | 5 |
| 2017 | sepsis symptoms | 5 |
| 2017 | blood infection | 5 |
| 2017 | septic | 5 |
| 2017 | sepse | 5 |
| 2017 | sepsis infection | 5 |
| 2017 | blood poisoning | 5 |
| 2017 | сепсис | 5 |
| 2017 | sepsi | 5 |
| 2017 | sepsis definition | 5 |
| 2017 | septicémie | 0 |
| 2017 | symptoms of sepsis | 0 |
| 2017 | pneumonia | 0 |
| 2017 | sepsis shock | 0 |
| 2017 | sepsis signs | 0 |
| 2017 | sepsis icd 10 | 0 |
| 2017 | setticemia | 0 |
| 2017 | septicaemia | 0 |
| 2017 | sepsis guidelines | 0 |
| 2017 | sepsis pneumonia | 0 |

**Table S4. Sepsis (Topic) Rising Related Queries Dataset**

| **Year** | **Query** | **ΔRSV** |
| --- | --- | --- |
| 2012 | 황수관 | ≥5000% |
| 2012 | caden beggan | 4400% |
| 2012 | savita halappanavar | 3400% |
| 2012 | 황수관 박사 | 2600% |
| 2012 | 급성 패혈증 | 650% |
| 2012 | world sepsis day | 650% |
| 2012 | 패혈증 이란 | 550% |
| 2012 | sepsa u noworodka | 190% |
| 2012 | surviving sepsis campaign 2012 | 140% |
| 2012 | surviving sepsis guidelines 2012 | 140% |
| 2012 | septic poisoning | 130% |
| 2012 | pneumococcal septicemia | 110% |
| 2012 | surviving sepsis 2012 | 110% |
| 2012 | tetanus | 100% |
| 2012 | icd 9 sepsis | 80% |
| 2012 | how do you get blood poisoning | 80% |
| 2012 | sceptical | 80% |
| 2012 | meningococcal septicaemia | 80% |
| 2012 | 패혈증 | 80% |
| 2012 | sepsis meaning | 70% |
| 2012 | septic shock definition | 70% |
| 2012 | septic shock คือ | 70% |
| 2012 | blodförgiftning symtom | 60% |
| 2012 | sepsis 2012 | 60% |
| 2012 | what is septicaemia | 60% |
| 2013 | 박용식 사망 패혈증 | ≥5000% |
| 2013 | sepsis guideline 2013 | ≥5000% |
| 2013 | 박용식 패혈증 으로 별세 | 4000% |
| 2013 | sepsis guidelines 2013 | 3750% |
| 2013 | posocznica u ludzi | 1000% |
| 2013 | sepsi infettiva | 750% |
| 2013 | 패혈증 증상 | 140% |
| 2013 | 敗血症 症狀 | 140% |
| 2013 | sobreviviendo a la sepsis 2012 | 120% |
| 2013 | sepse abdominal | 100% |
| 2013 | blood infection types | 100% |
| 2013 | co to jest sepsa | 90% |
| 2013 | icd 9 code for sepsis | 90% |
| 2013 | sepsis icd 9 | 80% |
| 2013 | late onset sepsis | 80% |
| 2013 | severe sepsis criteria | 80% |
| 2013 | icd 9 codes | 80% |
| 2013 | sepsis icd 9 code | 70% |
| 2013 | blodforgiftning symptomer | 60% |
| 2013 | sepsis meaning | 60% |
| 2013 | kriteria sepsis | 60% |
| 2013 | posocznica | 60% |
| 2013 | sobreviviendo a la sepsis | 60% |
| 2013 | symptome septicémie | 60% |
| 2013 | sepsis guideline 2012 | 60% |
| 2014 | sepsis guidelines 2014 | ≥5000% |
| 2014 | andressa urach | ≥5000% |
| 2014 | casey kasem | 3550% |
| 2014 | setticemia cause | 150% |
| 2014 | kaiser sepsis calculator | 150% |
| 2014 | сепсис симптомы | 110% |
| 2014 | sepsia significado | 100% |
| 2014 | sepsa u dziecka | 90% |
| 2014 | sepsis kills | 90% |
| 2014 | sepsis six | 80% |
| 2014 | objawy sepsy u dzieci | 70% |
| 2014 | 敗血症 と は | 70% |
| 2014 | 敗血症 症狀 | 70% |
| 2014 | cid sepse | 60% |
| 2014 | что такое сепсис | 60% |
| 2014 | what is septis | 60% |
| 2014 | septisemi nedir | 60% |
| 2014 | objawy sepsy | 60% |
| 2014 | o que é sepse | 50% |
| 2014 | sepsis définition | 50% |
| 2014 | sirs vs sepsis | 50% |
| 2014 | umbilical sepsis | 50% |
| 2014 | what is septic infection | 50% |
| 2014 | sepse grave | 50% |
| 2014 | sepsis definicion | 50% |
| 2015 | 河上 和雄 | 1650% |
| 2015 | sobreviviendo ala sepsis | 900% |
| 2015 | icd 10 code for sepsis | 450% |
| 2015 | sepsis icd 10 | 350% |
| 2015 | sepsi da meningococco | 200% |
| 2015 | sepsis hastalığı | 180% |
| 2015 | neisseria sepsis | 170% |
| 2015 | septicemia meaning | 160% |
| 2015 | septicemia definition | 140% |
| 2015 | sepsis alert | 140% |
| 2015 | что такое сепсис | 110% |
| 2015 | сепсис это | 100% |
| 2015 | 敗 血 病 | 90% |
| 2015 | septic workup | 80% |
| 2015 | cold sepsis | 70% |
| 2015 | was ist eine sepsis | 70% |
| 2015 | σηψη | 60% |
| 2015 | سپسیس | 60% |
| 2015 | kaiser sepsis calculator | 60% |
| 2015 | 敗血症 死亡率 | 60% |
| 2015 | 敗血症 症狀 | 50% |
| 2015 | sintomas de sepse | 50% |
| 2015 | 敗血症 症狀 | 50% |
| 2015 | septisemi | 40% |
| 2016 | patty duke | ≥5000% |
| 2016 | muhammad ali | ≥5000% |
| 2016 | muhammad ali death | ≥5000% |
| 2016 | surviving sepsis 2016 | ≥5000% |
| 2016 | sepsis guidelines 2016 | ≥5000% |
| 2016 | sepsis from a ruptured intestine | ≥5000% |
| 2016 | jama sepsis 2016 | ≥5000% |
| 2016 | sepsis ruptured intestine | ≥5000% |
| 2016 | surviving sepsis guidelines 2016 | ≥5000% |
| 2016 | patty duke death | 4800% |
| 2016 | how did muhammad ali die | 4250% |
| 2016 | patty duke died | 4250% |
| 2016 | sepsis-3 | 3600% |
| 2016 | qsofa | 3450% |
| 2016 | qsofa sepsis | 2850% |
| 2016 | mohamed ali | 2700% |
| 2016 | sepsis 2016 | 2050% |
| 2016 | sepsis guideline 2016 | 2000% |
| 2016 | sepsis criteria 2016 | 2000% |
| 2016 | sofa sepsis | 1300% |
| 2016 | jama sepsis | 1150% |
| 2016 | sofa | 1100% |
| 2016 | криптогенный сепсис | 1000% |
| 2016 | new sepsis definition | 750% |
| 2016 | sofa score sepsis | 750% |
| 2017 | posocznica plamista | ≥5000% |
| 2017 | 敗血症 っ て 何 | 4500% |
| 2017 | guillermo sanchez | 4300% |
| 2017 | sepsis guidelines 2017 | 2200% |
| 2017 | sepsis vitamin c | 2150% |
| 2017 | vitamin c for sepsis | 950% |
| 2017 | sepsis 2017 | 750% |
| 2017 | 敗 血 病 特效 藥 水果 | 500% |
| 2017 | what is sepsis in adults | 300% |
| 2017 | sepsi fulminante | 250% |
| 2017 | 패혈증 증상 | 200% |
| 2017 | meningokokna sepsa | 180% |
| 2017 | septicemia generalizada | 140% |
| 2017 | sepsa u dzieci objawy | 130% |
| 2017 | sepsis symptoms in adults | 120% |
| 2017 | symptoms of sepsis in adults | 120% |
| 2017 | sepsa u dziecka | 110% |
| 2017 | sepsis cure | 100% |
| 2017 | sepsis alert | 90% |
| 2017 | sepsis in adults | 80% |
| 2017 | sepsi osk | 80% |
| 2017 | whats sepsis | 80% |
| 2017 | 敗血症 と は | 70% |
| 2017 | 패혈증 이란 | 70% |
| 2017 | was ist eine sepsis | 70% |

**Table S5. Average Per-Country Sepsis (Topic) versus Malaria (Topic) Relative Search Volume Dataset**

| **ISO** | **Topic Code** | **Mean RSV** | **Year** |
| --- | --- | --- | --- |
| AD | Sepsis | 0 | 2007 - 2012 |
| AD | Malaria | 5.8544061 | 2007 - 2012 |
| AD | Sepsis | 0 | 2012 - 2017 |
| AD | Malaria | 5.7969349 | 2012 - 2017 |
| AE | Sepsis | 9.816092 | 2007 - 2012 |
| AE | Malaria | 32.386973 | 2007 - 2012 |
| AE | Sepsis | 29.819923 | 2012 - 2017 |
| AE | Malaria | 55.62069 | 2012 - 2017 |
| AF | Sepsis | 1.3908046 | 2007 - 2012 |
| AF | Malaria | 11.528736 | 2007 - 2012 |
| AF | Sepsis | 15.003831 | 2012 - 2017 |
| AF | Malaria | 31.206897 | 2012 - 2017 |
| AG | Sepsis | 7.7318008 | 2007 - 2012 |
| AG | Malaria | 12.268199 | 2007 - 2012 |
| AG | Sepsis | 9.2030651 | 2012 - 2017 |
| AG | Malaria | 7.5785441 | 2012 - 2017 |
| AL | Sepsis | 4.3908046 | 2007 - 2012 |
| AL | Malaria | 2.5632184 | 2007 - 2012 |
| AL | Sepsis | 21.245211 | 2012 - 2017 |
| AL | Malaria | 12.05364 | 2012 - 2017 |
| AM | Sepsis | 1.7624521 | 2007 - 2012 |
| AM | Malaria | 1.8505747 | 2007 - 2012 |
| AM | Sepsis | 18.961686 | 2012 - 2017 |
| AM | Malaria | 16.337165 | 2012 - 2017 |
| AO | Sepsis | 0.954023 | 2007 - 2012 |
| AO | Malaria | 14.402299 | 2007 - 2012 |
| AO | Sepsis | 4.2605364 | 2012 - 2017 |
| AO | Malaria | 40.961686 | 2012 - 2017 |
| AR | Sepsis | 17.072797 | 2007 - 2012 |
| AR | Malaria | 24.436782 | 2007 - 2012 |
| AR | Sepsis | 15.145594 | 2012 - 2017 |
| AR | Malaria | 14.022989 | 2012 - 2017 |
| AS | Sepsis | 0 | 2007 - 2012 |
| AS | Malaria | 3.8007663 | 2007 - 2012 |
| AS | Sepsis | 0 | 2012 - 2017 |
| AS | Malaria | 1.1187739 | 2012 - 2017 |
| AT | Sepsis | 35.812261 | 2007 - 2012 |
| AT | Malaria | 46.176245 | 2007 - 2012 |
| AT | Sepsis | 18.229885 | 2012 - 2017 |
| AT | Malaria | 17.91954 | 2012 - 2017 |
| AU | Sepsis | 26.222222 | 2007 - 2012 |
| AU | Malaria | 56.348659 | 2007 - 2012 |
| AU | Sepsis | 30.084291 | 2012 - 2017 |
| AU | Malaria | 37.222222 | 2012 - 2017 |
| AZ | Sepsis | 3.8390805 | 2007 - 2012 |
| AZ | Malaria | 6.559387 | 2007 - 2012 |
| AZ | Sepsis | 28.421456 | 2012 - 2017 |
| AZ | Malaria | 26.56705 | 2012 - 2017 |
| BA | Sepsis | 7.3524904 | 2007 - 2012 |
| BA | Malaria | 2.743295 | 2007 - 2012 |
| BA | Sepsis | 13.91954 | 2012 - 2017 |
| BA | Malaria | 7.0076628 | 2012 - 2017 |
| BB | Sepsis | 5.743295 | 2007 - 2012 |
| BB | Malaria | 8.51341 | 2007 - 2012 |
| BB | Sepsis | 7.1494253 | 2012 - 2017 |
| BB | Malaria | 6.9693487 | 2012 - 2017 |
| BD | Sepsis | 7.1264368 | 2007 - 2012 |
| BD | Malaria | 15.735632 | 2007 - 2012 |
| BD | Sepsis | 24.536398 | 2012 - 2017 |
| BD | Malaria | 37.67433 | 2012 - 2017 |
| BE | Sepsis | 17.624521 | 2007 - 2012 |
| BE | Malaria | 36.272031 | 2007 - 2012 |
| BE | Sepsis | 18.199234 | 2012 - 2017 |
| BE | Malaria | 28.938697 | 2012 - 2017 |
| BF | Sepsis | 0 | 2007 - 2012 |
| BF | Malaria | 14.314176 | 2007 - 2012 |
| BF | Sepsis | 0 | 2012 - 2017 |
| BF | Malaria | 39.950192 | 2012 - 2017 |
| BG | Sepsis | 13.632184 | 2007 - 2012 |
| BG | Malaria | 10.67433 | 2007 - 2012 |
| BG | Sepsis | 14.279693 | 2012 - 2017 |
| BG | Malaria | 13.67433 | 2012 - 2017 |
| BH | Sepsis | 7.1187739 | 2007 - 2012 |
| BH | Malaria | 16.088123 | 2007 - 2012 |
| BH | Sepsis | 22.019157 | 2012 - 2017 |
| BH | Malaria | 25.770115 | 2012 - 2017 |
| BI | Sepsis | 0 | 2007 - 2012 |
| BI | Malaria | 12.241379 | 2007 - 2012 |
| BI | Sepsis | 0 | 2012 - 2017 |
| BI | Malaria | 21.321839 | 2012 - 2017 |
| BJ | Sepsis | 0 | 2007 - 2012 |
| BJ | Malaria | 16.16092 | 2007 - 2012 |
| BJ | Sepsis | 0 | 2012 - 2017 |
| BJ | Malaria | 31.988506 | 2012 - 2017 |
| BN | Sepsis | 2.9463602 | 2007 - 2012 |
| BN | Malaria | 7.1877395 | 2007 - 2012 |
| BN | Sepsis | 10.896552 | 2012 - 2017 |
| BN | Malaria | 21.781609 | 2012 - 2017 |
| BO | Sepsis | 14.065134 | 2007 - 2012 |
| BO | Malaria | 21.693487 | 2007 - 2012 |
| BO | Sepsis | 33.019157 | 2012 - 2017 |
| BO | Malaria | 44.509579 | 2012 - 2017 |
| BR | Sepsis | 18.340996 | 2007 - 2012 |
| BR | Malaria | 43.670498 | 2007 - 2012 |
| BR | Sepsis | 10.417625 | 2012 - 2017 |
| BR | Malaria | 16.068966 | 2012 - 2017 |
| BS | Sepsis | 5.6934866 | 2007 - 2012 |
| BS | Malaria | 11.302682 | 2007 - 2012 |
| BS | Sepsis | 8.9386973 | 2012 - 2017 |
| BS | Malaria | 5.8735632 | 2012 - 2017 |
| BT | Sepsis | 1.2950192 | 2007 - 2012 |
| BT | Malaria | 4.8199234 | 2007 - 2012 |
| BT | Sepsis | 8.2490421 | 2012 - 2017 |
| BT | Malaria | 17.279693 | 2012 - 2017 |
| BW | Sepsis | 2.467433 | 2007 - 2012 |
| BW | Malaria | 11.363985 | 2007 - 2012 |
| BW | Sepsis | 5.4214559 | 2012 - 2017 |
| BW | Malaria | 17.574713 | 2012 - 2017 |
| BY | Sepsis | 11.375479 | 2007 - 2012 |
| BY | Malaria | 7.3716475 | 2007 - 2012 |
| BY | Sepsis | 28.827586 | 2012 - 2017 |
| BY | Malaria | 18.390805 | 2012 - 2017 |
| BZ | Sepsis | 4.5172414 | 2007 - 2012 |
| BZ | Malaria | 14.835249 | 2007 - 2012 |
| BZ | Sepsis | 8.3218391 | 2012 - 2017 |
| BZ | Malaria | 23.793103 | 2012 - 2017 |
| CA | Sepsis | 23.727969 | 2007 - 2012 |
| CA | Malaria | 39.816092 | 2007 - 2012 |
| CA | Sepsis | 30.597701 | 2012 - 2017 |
| CA | Malaria | 28.816092 | 2012 - 2017 |
| CD | Sepsis | 1.1455939 | 2007 - 2012 |
| CD | Malaria | 12.697318 | 2007 - 2012 |
| CD | Sepsis | 7.908046 | 2012 - 2017 |
| CD | Malaria | 51.662835 | 2012 - 2017 |
| CF | Sepsis | 0 | 2007 - 2012 |
| CF | Malaria | 2.1111111 | 2007 - 2012 |
| CF | Sepsis | 0 | 2012 - 2017 |
| CF | Malaria | 11.965517 | 2012 - 2017 |
| CG | Sepsis | 0.2183908 | 2007 - 2012 |
| CG | Malaria | 3.4521073 | 2007 - 2012 |
| CG | Sepsis | 2.5823755 | 2012 - 2017 |
| CG | Malaria | 28.632184 | 2012 - 2017 |
| CH | Sepsis | 13.16092 | 2007 - 2012 |
| CH | Malaria | 30.272031 | 2007 - 2012 |
| CH | Sepsis | 22.149425 | 2012 - 2017 |
| CH | Malaria | 34.386973 | 2012 - 2017 |
| CI | Sepsis | 1.0498084 | 2007 - 2012 |
| CI | Malaria | 22.295019 | 2007 - 2012 |
| CI | Sepsis | 2.835249 | 2012 - 2017 |
| CI | Malaria | 44.885057 | 2012 - 2017 |
| CL | Sepsis | 25.547893 | 2007 - 2012 |
| CL | Malaria | 20.56705 | 2007 - 2012 |
| CL | Sepsis | 5.5057471 | 2012 - 2017 |
| CL | Malaria | 3.4329502 | 2012 - 2017 |
| CM | Sepsis | 0.954023 | 2007 - 2012 |
| CM | Malaria | 20.478927 | 2007 - 2012 |
| CM | Sepsis | 4.4367816 | 2012 - 2017 |
| CM | Malaria | 45.735632 | 2012 - 2017 |
| CN | Sepsis | 15.222222 | 2007 - 2012 |
| CN | Malaria | 14.32567 | 2007 - 2012 |
| CN | Sepsis | 8.2490421 | 2012 - 2017 |
| CN | Malaria | 14.038314 | 2012 - 2017 |
| CO | Sepsis | 21 | 2007 - 2012 |
| CO | Malaria | 43.214559 | 2007 - 2012 |
| CO | Sepsis | 40.628352 | 2012 - 2017 |
| CO | Malaria | 62.145594 | 2012 - 2017 |
| CR | Sepsis | 10.911877 | 2007 - 2012 |
| CR | Malaria | 23.954023 | 2007 - 2012 |
| CR | Sepsis | 14.114943 | 2012 - 2017 |
| CR | Malaria | 19.831418 | 2012 - 2017 |
| CU | Sepsis | 29.157088 | 2007 - 2012 |
| CU | Malaria | 19.226054 | 2007 - 2012 |
| CU | Sepsis | 9.1340996 | 2012 - 2017 |
| CU | Malaria | 8.789272 | 2012 - 2017 |
| CV | Sepsis | 0 | 2007 - 2012 |
| CV | Malaria | 2.7624521 | 2007 - 2012 |
| CV | Sepsis | 3.8773946 | 2012 - 2017 |
| CV | Malaria | 17.329502 | 2012 - 2017 |
| CW | Sepsis | 9.9731801 | 2012 - 2017 |
| CW | Malaria | 11.130268 | 2012 - 2017 |
| CY | Sepsis | 5.1034483 | 2007 - 2012 |
| CY | Malaria | 10.636015 | 2007 - 2012 |
| CY | Sepsis | 4.8467433 | 2012 - 2017 |
| CY | Malaria | 5.5478927 | 2012 - 2017 |
| CZ | Sepsis | 16.766284 | 2007 - 2012 |
| CZ | Malaria | 18.413793 | 2007 - 2012 |
| CZ | Sepsis | 6.6436782 | 2012 - 2017 |
| CZ | Malaria | 5.3448276 | 2012 - 2017 |
| DE | Sepsis | 21.831418 | 2007 - 2012 |
| DE | Malaria | 29.743295 | 2007 - 2012 |
| DE | Sepsis | 55.67433 | 2012 - 2017 |
| DE | Malaria | 51.318008 | 2012 - 2017 |
| DJ | Sepsis | 0 | 2007 - 2012 |
| DJ | Malaria | 4.6819923 | 2007 - 2012 |
| DJ | Sepsis | 0 | 2012 - 2017 |
| DJ | Malaria | 17.003831 | 2012 - 2017 |
| DK | Sepsis | 16.467433 | 2007 - 2012 |
| DK | Malaria | 32.398467 | 2007 - 2012 |
| DK | Sepsis | 36.992337 | 2012 - 2017 |
| DK | Malaria | 48.306513 | 2012 - 2017 |
| DM | Sepsis | 5.4750958 | 2007 - 2012 |
| DM | Malaria | 4.1724138 | 2007 - 2012 |
| DM | Sepsis | 11.670498 | 2012 - 2017 |
| DM | Malaria | 12.777778 | 2012 - 2017 |
| DO | Sepsis | 10.413793 | 2007 - 2012 |
| DO | Malaria | 19.636015 | 2007 - 2012 |
| DO | Sepsis | 7.4061303 | 2012 - 2017 |
| DO | Malaria | 13.678161 | 2012 - 2017 |
| DZ | Sepsis | 7.2796935 | 2007 - 2012 |
| DZ | Malaria | 15.229885 | 2007 - 2012 |
| DZ | Sepsis | 1.1724138 | 2012 - 2017 |
| DZ | Malaria | 3.4444444 | 2012 - 2017 |
| EC | Sepsis | 14.915709 | 2007 - 2012 |
| EC | Malaria | 27.605364 | 2007 - 2012 |
| EC | Sepsis | 38.850575 | 2012 - 2017 |
| EC | Malaria | 43.609195 | 2012 - 2017 |
| EE | Sepsis | 13.992337 | 2007 - 2012 |
| EE | Malaria | 19.145594 | 2007 - 2012 |
| EE | Sepsis | 6.5823755 | 2012 - 2017 |
| EE | Malaria | 6.605364 | 2012 - 2017 |
| EG | Sepsis | 20.731801 | 2007 - 2012 |
| EG | Malaria | 25.62069 | 2007 - 2012 |
| EG | Sepsis | 7.4559387 | 2012 - 2017 |
| EG | Malaria | 7.5402299 | 2012 - 2017 |
| ER | Sepsis | 0 | 2007 - 2012 |
| ER | Malaria | 9.1302682 | 2007 - 2012 |
| ER | Sepsis | 0 | 2012 - 2017 |
| ER | Malaria | 7.7318008 | 2012 - 2017 |
| ES | Sepsis | 4.091954 | 2007 - 2012 |
| ES | Malaria | 8.0153257 | 2007 - 2012 |
| ES | Sepsis | 14.049808 | 2012 - 2017 |
| ES | Malaria | 20.509579 | 2012 - 2017 |
| ET | Sepsis | 1.8237548 | 2007 - 2012 |
| ET | Malaria | 17.597701 | 2007 - 2012 |
| ET | Sepsis | 11.222222 | 2012 - 2017 |
| ET | Malaria | 44.850575 | 2012 - 2017 |
| FI | Sepsis | 7.7471264 | 2007 - 2012 |
| FI | Malaria | 8.9348659 | 2007 - 2012 |
| FI | Sepsis | 26.153257 | 2012 - 2017 |
| FI | Malaria | 19.908046 | 2012 - 2017 |
| FJ | Sepsis | 3.4942529 | 2007 - 2012 |
| FJ | Malaria | 3.6398467 | 2007 - 2012 |
| FJ | Sepsis | 20.065134 | 2012 - 2017 |
| FJ | Malaria | 17.689655 | 2012 - 2017 |
| FR | Sepsis | 17.628352 | 2007 - 2012 |
| FR | Malaria | 51.149425 | 2007 - 2012 |
| FR | Sepsis | 16.114943 | 2012 - 2017 |
| FR | Malaria | 30.10728 | 2012 - 2017 |
| GA | Sepsis | 0 | 2007 - 2012 |
| GA | Malaria | 12.02682 | 2007 - 2012 |
| GA | Sepsis | 0 | 2012 - 2017 |
| GA | Malaria | 32.659004 | 2012 - 2017 |
| GB | Sepsis | 8.6781609 | 2007 - 2012 |
| GB | Malaria | 20.007663 | 2007 - 2012 |
| GB | Sepsis | 15.727969 | 2012 - 2017 |
| GB | Malaria | 12.666667 | 2012 - 2017 |
| GD | Sepsis | 5.3065134 | 2007 - 2012 |
| GD | Malaria | 5.8888889 | 2007 - 2012 |
| GD | Sepsis | 8.7471264 | 2012 - 2017 |
| GD | Malaria | 7.7318008 | 2012 - 2017 |
| GE | Sepsis | 3.8122605 | 2007 - 2012 |
| GE | Malaria | 3.2068966 | 2007 - 2012 |
| GE | Sepsis | 28.490421 | 2012 - 2017 |
| GE | Malaria | 15.390805 | 2012 - 2017 |
| GF | Sepsis | 0 | 2007 - 2012 |
| GF | Malaria | 4.8773946 | 2007 - 2012 |
| GF | Sepsis | 0 | 2012 - 2017 |
| GF | Malaria | 13.498084 | 2012 - 2017 |
| GG | Sepsis | 3.9693487 | 2007 - 2012 |
| GG | Malaria | 0 | 2007 - 2012 |
| GG | Sepsis | 16.183908 | 2012 - 2017 |
| GG | Malaria | 0 | 2012 - 2017 |
| GH | Sepsis | 3.3103448 | 2007 - 2012 |
| GH | Malaria | 35.199234 | 2007 - 2012 |
| GH | Sepsis | 7.9195402 | 2012 - 2017 |
| GH | Malaria | 58.283525 | 2012 - 2017 |
| GI | Sepsis | 4.0996169 | 2007 - 2012 |
| GI | Malaria | 4.3869732 | 2007 - 2012 |
| GI | Sepsis | 8.9655172 | 2012 - 2017 |
| GI | Malaria | 7.9386973 | 2012 - 2017 |
| GM | Sepsis | 0 | 2007 - 2012 |
| GM | Malaria | 17.662835 | 2007 - 2012 |
| GM | Sepsis | 0 | 2012 - 2017 |
| GM | Malaria | 22.452107 | 2012 - 2017 |
| GN | Sepsis | 0.0613027 | 2007 - 2012 |
| GN | Malaria | 8.816092 | 2007 - 2012 |
| GN | Sepsis | 0.5019157 | 2012 - 2017 |
| GN | Malaria | 10.501916 | 2012 - 2017 |
| GP | Sepsis | 3.4559387 | 2007 - 2012 |
| GP | Malaria | 3.7624521 | 2007 - 2012 |
| GP | Sepsis | 16.881226 | 2012 - 2017 |
| GP | Malaria | 18.214559 | 2012 - 2017 |
| GQ | Sepsis | 0 | 2007 - 2012 |
| GQ | Malaria | 4 | 2007 - 2012 |
| GQ | Sepsis | 0 | 2012 - 2017 |
| GQ | Malaria | 24.812261 | 2012 - 2017 |
| GR | Sepsis | 18.429119 | 2007 - 2012 |
| GR | Malaria | 19.444444 | 2007 - 2012 |
| GR | Sepsis | 2.467433 | 2012 - 2017 |
| GR | Malaria | 2.4252874 | 2012 - 2017 |
| GT | Sepsis | 11.609195 | 2007 - 2012 |
| GT | Malaria | 22.283525 | 2007 - 2012 |
| GT | Sepsis | 19.218391 | 2012 - 2017 |
| GT | Malaria | 31.042146 | 2012 - 2017 |
| GU | Sepsis | 8.0613027 | 2007 - 2012 |
| GU | Malaria | 7.8773946 | 2007 - 2012 |
| GU | Sepsis | 15.731801 | 2012 - 2017 |
| GU | Malaria | 11.042146 | 2012 - 2017 |
| GW | Sepsis | 0 | 2007 - 2012 |
| GW | Malaria | 1.1111111 | 2007 - 2012 |
| GW | Sepsis | 0 | 2012 - 2017 |
| GW | Malaria | 12.436782 | 2012 - 2017 |
| GY | Sepsis | 2.9616858 | 2007 - 2012 |
| GY | Malaria | 13.574713 | 2007 - 2012 |
| GY | Sepsis | 7.3793103 | 2012 - 2017 |
| GY | Malaria | 22.869732 | 2012 - 2017 |
| HK | Sepsis | 13.800766 | 2007 - 2012 |
| HK | Malaria | 25.302682 | 2007 - 2012 |
| HK | Sepsis | 12.555556 | 2012 - 2017 |
| HK | Malaria | 14.555556 | 2012 - 2017 |
| HN | Sepsis | 5.4597701 | 2007 - 2012 |
| HN | Malaria | 19.045977 | 2007 - 2012 |
| HN | Sepsis | 17.183908 | 2012 - 2017 |
| HN | Malaria | 33.735632 | 2012 - 2017 |
| HR | Sepsis | 10.586207 | 2007 - 2012 |
| HR | Malaria | 5.6743295 | 2007 - 2012 |
| HR | Sepsis | 14.743295 | 2012 - 2017 |
| HR | Malaria | 6.4750958 | 2012 - 2017 |
| HT | Sepsis | 1.4367816 | 2007 - 2012 |
| HT | Malaria | 21.034483 | 2007 - 2012 |
| HT | Sepsis | 8.532567 | 2012 - 2017 |
| HT | Malaria | 35.708812 | 2012 - 2017 |
| HU | Sepsis | 30.421456 | 2007 - 2012 |
| HU | Malaria | 22.681992 | 2007 - 2012 |
| HU | Sepsis | 52.122605 | 2012 - 2017 |
| HU | Malaria | 25.233716 | 2012 - 2017 |
| ID | Sepsis | 10.329502 | 2007 - 2012 |
| ID | Malaria | 42.816092 | 2007 - 2012 |
| ID | Sepsis | 18.789272 | 2012 - 2017 |
| ID | Malaria | 64.804598 | 2012 - 2017 |
| IE | Sepsis | 7.0574713 | 2007 - 2012 |
| IE | Malaria | 9.6704981 | 2007 - 2012 |
| IE | Sepsis | 26.666667 | 2012 - 2017 |
| IE | Malaria | 15.249042 | 2012 - 2017 |
| IL | Sepsis | 15.773946 | 2007 - 2012 |
| IL | Malaria | 31.291188 | 2007 - 2012 |
| IL | Sepsis | 17.547893 | 2012 - 2017 |
| IL | Malaria | 27.367816 | 2012 - 2017 |
| IM | Sepsis | 2.4176245 | 2007 - 2012 |
| IM | Malaria | 3.3180077 | 2007 - 2012 |
| IM | Sepsis | 18.455939 | 2012 - 2017 |
| IM | Malaria | 10.05364 | 2012 - 2017 |
| IN | Sepsis | 19.084291 | 2007 - 2012 |
| IN | Malaria | 49.37931 | 2007 - 2012 |
| IN | Sepsis | 18.471264 | 2012 - 2017 |
| IN | Malaria | 58.984674 | 2012 - 2017 |
| IQ | Sepsis | 2.5670498 | 2007 - 2012 |
| IQ | Malaria | 6.7931034 | 2007 - 2012 |
| IQ | Sepsis | 19.038314 | 2012 - 2017 |
| IQ | Malaria | 26.965517 | 2012 - 2017 |
| IR | Sepsis | 11.632184 | 2007 - 2012 |
| IR | Malaria | 26.509579 | 2007 - 2012 |
| IR | Sepsis | 20.59387 | 2012 - 2017 |
| IR | Malaria | 45.655172 | 2012 - 2017 |
| IS | Sepsis | 17.731801 | 2007 - 2012 |
| IS | Malaria | 21.494253 | 2007 - 2012 |
| IS | Sepsis | 20.084291 | 2012 - 2017 |
| IS | Malaria | 20.011494 | 2012 - 2017 |
| IT | Sepsis | 6.954023 | 2007 - 2012 |
| IT | Malaria | 9.0268199 | 2007 - 2012 |
| IT | Sepsis | 34.444444 | 2012 - 2017 |
| IT | Malaria | 29.137931 | 2012 - 2017 |
| JE | Sepsis | 2.3218391 | 2007 - 2012 |
| JE | Malaria | 7.6819923 | 2007 - 2012 |
| JE | Sepsis | 17.846743 | 2012 - 2017 |
| JE | Malaria | 19.51341 | 2012 - 2017 |
| JM | Sepsis | 7.7547893 | 2007 - 2012 |
| JM | Malaria | 20.988506 | 2007 - 2012 |
| JM | Sepsis | 15 | 2012 - 2017 |
| JM | Malaria | 17.421456 | 2012 - 2017 |
| JO | Sepsis | 8.9616858 | 2007 - 2012 |
| JO | Malaria | 13.111111 | 2007 - 2012 |
| JO | Sepsis | 15.83908 | 2012 - 2017 |
| JO | Malaria | 16.340996 | 2012 - 2017 |
| JP | Sepsis | 8.5095785 | 2007 - 2012 |
| JP | Malaria | 6.0574713 | 2007 - 2012 |
| JP | Sepsis | 23.685824 | 2012 - 2017 |
| JP | Malaria | 10.302682 | 2012 - 2017 |
| KE | Sepsis | 4.0651341 | 2007 - 2012 |
| KE | Malaria | 37.034483 | 2007 - 2012 |
| KE | Sepsis | 9.6819923 | 2012 - 2017 |
| KE | Malaria | 52.37931 | 2012 - 2017 |
| KG | Sepsis | 2.6321839 | 2007 - 2012 |
| KG | Malaria | 5.1685824 | 2007 - 2012 |
| KG | Sepsis | 18.681992 | 2012 - 2017 |
| KG | Malaria | 16.176245 | 2012 - 2017 |
| KH | Sepsis | 0.7701149 | 2007 - 2012 |
| KH | Malaria | 15.670498 | 2007 - 2012 |
| KH | Sepsis | 6.9425287 | 2012 - 2017 |
| KH | Malaria | 44.195402 | 2012 - 2017 |
| KR | Sepsis | 16.873563 | 2007 - 2012 |
| KR | Malaria | 16.735632 | 2007 - 2012 |
| KR | Sepsis | 5.1762452 | 2012 - 2017 |
| KR | Malaria | 2.6704981 | 2012 - 2017 |
| KW | Sepsis | 8.789272 | 2007 - 2012 |
| KW | Malaria | 15.586207 | 2007 - 2012 |
| KW | Sepsis | 18.517241 | 2012 - 2017 |
| KW | Malaria | 22.249042 | 2012 - 2017 |
| KY | Sepsis | 6.3103448 | 2007 - 2012 |
| KY | Malaria | 7.559387 | 2007 - 2012 |
| KY | Sepsis | 13.321839 | 2012 - 2017 |
| KY | Malaria | 13.586207 | 2012 - 2017 |
| KZ | Sepsis | 5.954023 | 2007 - 2012 |
| KZ | Malaria | 5.0689655 | 2007 - 2012 |
| KZ | Sepsis | 22.869732 | 2012 - 2017 |
| KZ | Malaria | 14.195402 | 2012 - 2017 |
| LA | Sepsis | 1.210728 | 2007 - 2012 |
| LA | Malaria | 10.881226 | 2007 - 2012 |
| LA | Sepsis | 9.8697318 | 2012 - 2017 |
| LA | Malaria | 38.720307 | 2012 - 2017 |
| LB | Sepsis | 7.5287356 | 2007 - 2012 |
| LB | Malaria | 17.375479 | 2007 - 2012 |
| LB | Sepsis | 20.030651 | 2012 - 2017 |
| LB | Malaria | 29.731801 | 2012 - 2017 |
| LC | Sepsis | 0.3793103 | 2007 - 2012 |
| LC | Malaria | 1.2988506 | 2007 - 2012 |
| LC | Sepsis | 7.0842912 | 2012 - 2017 |
| LC | Malaria | 8.1724138 | 2012 - 2017 |
| LK | Sepsis | 4.3218391 | 2007 - 2012 |
| LK | Malaria | 16.97318 | 2007 - 2012 |
| LK | Sepsis | 27.965517 | 2012 - 2017 |
| LK | Malaria | 45.835249 | 2012 - 2017 |
| LR | Sepsis | 0.0689655 | 2007 - 2012 |
| LR | Malaria | 3.7816092 | 2007 - 2012 |
| LR | Sepsis | 1.7203065 | 2012 - 2017 |
| LR | Malaria | 14.643678 | 2012 - 2017 |
| LS | Sepsis | 0 | 2007 - 2012 |
| LS | Malaria | 1.9501916 | 2007 - 2012 |
| LS | Sepsis | 0 | 2012 - 2017 |
| LS | Malaria | 3.4827586 | 2012 - 2017 |
| LT | Sepsis | 7.9425287 | 2007 - 2012 |
| LT | Malaria | 6.6743295 | 2007 - 2012 |
| LT | Sepsis | 11.360153 | 2012 - 2017 |
| LT | Malaria | 5.7547893 | 2012 - 2017 |
| LU | Sepsis | 5.3601533 | 2007 - 2012 |
| LU | Malaria | 15.877395 | 2007 - 2012 |
| LU | Sepsis | 15.321839 | 2012 - 2017 |
| LU | Malaria | 23.938697 | 2012 - 2017 |
| LV | Sepsis | 4.9923372 | 2007 - 2012 |
| LV | Malaria | 6.9195402 | 2007 - 2012 |
| LV | Sepsis | 2.2988506 | 2012 - 2017 |
| LV | Malaria | 1.5747126 | 2012 - 2017 |
| LY | Sepsis | 0.4367816 | 2007 - 2012 |
| LY | Malaria | 1.4482759 | 2007 - 2012 |
| LY | Sepsis | 8.5057471 | 2012 - 2017 |
| LY | Malaria | 16.83908 | 2012 - 2017 |
| MA | Sepsis | 6.5900383 | 2007 - 2012 |
| MA | Malaria | 19.409962 | 2007 - 2012 |
| MA | Sepsis | 21.168582 | 2012 - 2017 |
| MA | Malaria | 51 | 2012 - 2017 |
| MD | Sepsis | 6.6091954 | 2007 - 2012 |
| MD | Malaria | 6.862069 | 2007 - 2012 |
| MD | Sepsis | 30.605364 | 2012 - 2017 |
| MD | Malaria | 25.35249 | 2012 - 2017 |
| ME | Sepsis | 2.8390805 | 2007 - 2012 |
| ME | Malaria | 2.2643678 | 2007 - 2012 |
| ME | Sepsis | 15.938697 | 2012 - 2017 |
| ME | Malaria | 8.8927203 | 2012 - 2017 |
| MG | Sepsis | 1.2643678 | 2007 - 2012 |
| MG | Malaria | 18.628352 | 2007 - 2012 |
| MG | Sepsis | 2.7011494 | 2012 - 2017 |
| MG | Malaria | 38.16092 | 2012 - 2017 |
| MK | Sepsis | 6.2950192 | 2007 - 2012 |
| MK | Malaria | 4.0383142 | 2007 - 2012 |
| MK | Sepsis | 12.421456 | 2012 - 2017 |
| MK | Malaria | 9.8467433 | 2012 - 2017 |
| ML | Sepsis | 1.091954 | 2007 - 2012 |
| ML | Malaria | 21.885057 | 2007 - 2012 |
| ML | Sepsis | 6.8275862 | 2012 - 2017 |
| ML | Malaria | 37.344828 | 2012 - 2017 |
| MM | Sepsis | 1.6704981 | 2007 - 2012 |
| MM | Malaria | 9.8735632 | 2007 - 2012 |
| MM | Sepsis | 10.011494 | 2012 - 2017 |
| MM | Malaria | 33.678161 | 2012 - 2017 |
| MN | Sepsis | 1.6475096 | 2007 - 2012 |
| MN | Malaria | 3.045977 | 2007 - 2012 |
| MN | Sepsis | 15.743295 | 2012 - 2017 |
| MN | Malaria | 12.563218 | 2012 - 2017 |
| MO | Sepsis | 5.5862069 | 2007 - 2012 |
| MO | Malaria | 7.2030651 | 2007 - 2012 |
| MO | Sepsis | 18.724138 | 2012 - 2017 |
| MO | Malaria | 15.095785 | 2012 - 2017 |
| MQ | Sepsis | 2.743295 | 2007 - 2012 |
| MQ | Malaria | 6.2873563 | 2007 - 2012 |
| MQ | Sepsis | 7.6858238 | 2012 - 2017 |
| MQ | Malaria | 12.796935 | 2012 - 2017 |
| MR | Sepsis | 0 | 2007 - 2012 |
| MR | Malaria | 11.724138 | 2007 - 2012 |
| MR | Sepsis | 0 | 2012 - 2017 |
| MR | Malaria | 16.429119 | 2012 - 2017 |
| MT | Sepsis | 5.8045977 | 2007 - 2012 |
| MT | Malaria | 10.789272 | 2007 - 2012 |
| MT | Sepsis | 12.176245 | 2012 - 2017 |
| MT | Malaria | 14.329502 | 2012 - 2017 |
| MU | Sepsis | 3.302682 | 2007 - 2012 |
| MU | Malaria | 10.444444 | 2007 - 2012 |
| MU | Sepsis | 14.494253 | 2012 - 2017 |
| MU | Malaria | 25.118774 | 2012 - 2017 |
| MV | Sepsis | 2.1800766 | 2007 - 2012 |
| MV | Malaria | 4.1340996 | 2007 - 2012 |
| MV | Sepsis | 10.436782 | 2012 - 2017 |
| MV | Malaria | 17.478927 | 2012 - 2017 |
| MW | Sepsis | 1.8735632 | 2007 - 2012 |
| MW | Malaria | 15.333333 | 2007 - 2012 |
| MW | Sepsis | 9.3984674 | 2012 - 2017 |
| MW | Malaria | 36.367816 | 2012 - 2017 |
| MX | Sepsis | 39.279693 | 2007 - 2012 |
| MX | Malaria | 37.302682 | 2007 - 2012 |
| MX | Sepsis | 41.183908 | 2012 - 2017 |
| MX | Malaria | 30.816092 | 2012 - 2017 |
| MY | Sepsis | 27.022989 | 2007 - 2012 |
| MY | Malaria | 45.314176 | 2007 - 2012 |
| MY | Sepsis | 20.869732 | 2012 - 2017 |
| MY | Malaria | 23.452107 | 2012 - 2017 |
| MZ | Sepsis | 2.0536398 | 2007 - 2012 |
| MZ | Malaria | 29.32567 | 2007 - 2012 |
| MZ | Sepsis | 4.2145594 | 2012 - 2017 |
| MZ | Malaria | 46.195402 | 2012 - 2017 |
| NA | Sepsis | 35.938697 | 2007 - 2012 |
| NA | Malaria | 55.547893 | 2007 - 2012 |
| NA | Sepsis | 51.509579 | 2012 - 2017 |
| NA | Malaria | 57.394636 | 2012 - 2017 |
| NC | Sepsis | 1.9272031 | 2007 - 2012 |
| NC | Malaria | 6.1072797 | 2007 - 2012 |
| NC | Sepsis | 11.429119 | 2012 - 2017 |
| NC | Malaria | 19.130268 | 2012 - 2017 |
| NE | Sepsis | 0.0498084 | 2007 - 2012 |
| NE | Malaria | 5.1877395 | 2007 - 2012 |
| NE | Sepsis | 1.2681992 | 2012 - 2017 |
| NE | Malaria | 17.226054 | 2012 - 2017 |
| NG | Sepsis | 4.2758621 | 2007 - 2012 |
| NG | Malaria | 38.111111 | 2007 - 2012 |
| NG | Sepsis | 6.4176245 | 2012 - 2017 |
| NG | Malaria | 69.43295 | 2012 - 2017 |
| NI | Sepsis | 4.9386973 | 2007 - 2012 |
| NI | Malaria | 10.191571 | 2007 - 2012 |
| NI | Sepsis | 16.394636 | 2012 - 2017 |
| NI | Malaria | 23.789272 | 2012 - 2017 |
| NL | Sepsis | 18.356322 | 2007 - 2012 |
| NL | Malaria | 44.896552 | 2007 - 2012 |
| NL | Sepsis | 33.153257 | 2012 - 2017 |
| NL | Malaria | 63.084291 | 2012 - 2017 |
| NO | Sepsis | 24.249042 | 2007 - 2012 |
| NO | Malaria | 36.222222 | 2007 - 2012 |
| NO | Sepsis | 31.498084 | 2012 - 2017 |
| NO | Malaria | 26.965517 | 2012 - 2017 |
| NP | Sepsis | 3.7279693 | 2007 - 2012 |
| NP | Malaria | 11.045977 | 2007 - 2012 |
| NP | Sepsis | 23.701149 | 2012 - 2017 |
| NP | Malaria | 37.758621 | 2012 - 2017 |
| NZ | Sepsis | 25.471264 | 2007 - 2012 |
| NZ | Malaria | 36.226054 | 2007 - 2012 |
| NZ | Sepsis | 31.034483 | 2012 - 2017 |
| NZ | Malaria | 30.314176 | 2012 - 2017 |
| OM | Sepsis | 5.1724138 | 2007 - 2012 |
| OM | Malaria | 16.210728 | 2007 - 2012 |
| OM | Sepsis | 26.252874 | 2012 - 2017 |
| OM | Malaria | 42.145594 | 2012 - 2017 |
| PA | Sepsis | 7.9157088 | 2007 - 2012 |
| PA | Malaria | 14.731801 | 2007 - 2012 |
| PA | Sepsis | 7.0076628 | 2012 - 2017 |
| PA | Malaria | 8.2030651 | 2012 - 2017 |
| PE | Sepsis | 25.409962 | 2007 - 2012 |
| PE | Malaria | 42 | 2007 - 2012 |
| PE | Sepsis | 29.731801 | 2012 - 2017 |
| PE | Malaria | 34.444444 | 2012 - 2017 |
| PF | Sepsis | 3.7547893 | 2007 - 2012 |
| PF | Malaria | 6.3601533 | 2007 - 2012 |
| PF | Sepsis | 3.0191571 | 2012 - 2017 |
| PF | Malaria | 6.5862069 | 2012 - 2017 |
| PG | Sepsis | 0.3754789 | 2007 - 2012 |
| PG | Malaria | 7.8122605 | 2007 - 2012 |
| PG | Sepsis | 3.0766284 | 2012 - 2017 |
| PG | Malaria | 17.559387 | 2012 - 2017 |
| PH | Sepsis | 27.022989 | 2007 - 2012 |
| PH | Malaria | 29.417625 | 2007 - 2012 |
| PH | Sepsis | 38.40613 | 2012 - 2017 |
| PH | Malaria | 32.440613 | 2012 - 2017 |
| PK | Sepsis | 13.551724 | 2007 - 2012 |
| PK | Malaria | 32.762452 | 2007 - 2012 |
| PK | Sepsis | 19.693487 | 2012 - 2017 |
| PK | Malaria | 55.881226 | 2012 - 2017 |
| PL | Sepsis | 4.4444444 | 2007 - 2012 |
| PL | Malaria | 1.2873563 | 2007 - 2012 |
| PL | Sepsis | 27.547893 | 2012 - 2017 |
| PL | Malaria | 10.015326 | 2012 - 2017 |
| PR | Sepsis | 11.570881 | 2007 - 2012 |
| PR | Malaria | 13.275862 | 2007 - 2012 |
| PR | Sepsis | 1.6704981 | 2012 - 2017 |
| PR | Malaria | 1.7318008 | 2012 - 2017 |
| PS | Sepsis | 3.2950192 | 2007 - 2012 |
| PS | Malaria | 7.6206897 | 2007 - 2012 |
| PS | Sepsis | 5.0651341 | 2012 - 2017 |
| PS | Malaria | 7.2605364 | 2012 - 2017 |
| PT | Sepsis | 9.8237548 | 2007 - 2012 |
| PT | Malaria | 12.785441 | 2007 - 2012 |
| PT | Sepsis | 12.402299 | 2012 - 2017 |
| PT | Malaria | 18.942529 | 2012 - 2017 |
| PY | Sepsis | 7.4099617 | 2007 - 2012 |
| PY | Malaria | 8.5555556 | 2007 - 2012 |
| PY | Sepsis | 14.034483 | 2012 - 2017 |
| PY | Malaria | 11.773946 | 2012 - 2017 |
| QA | Sepsis | 6.5670498 | 2007 - 2012 |
| QA | Malaria | 16.816092 | 2007 - 2012 |
| QA | Sepsis | 27.747126 | 2012 - 2017 |
| QA | Malaria | 36.586207 | 2012 - 2017 |
| RE | Sepsis | 1.6628352 | 2007 - 2012 |
| RE | Malaria | 12.616858 | 2007 - 2012 |
| RE | Sepsis | 19.256705 | 2012 - 2017 |
| RE | Malaria | 34.632184 | 2012 - 2017 |
| RO | Sepsis | 10.793103 | 2007 - 2012 |
| RO | Malaria | 7.48659 | 2007 - 2012 |
| RO | Sepsis | 11.233716 | 2012 - 2017 |
| RO | Malaria | 7.3256705 | 2012 - 2017 |
| RS | Sepsis | 14.678161 | 2007 - 2012 |
| RS | Malaria | 10.666667 | 2007 - 2012 |
| RS | Sepsis | 13.333333 | 2012 - 2017 |
| RS | Malaria | 6.605364 | 2012 - 2017 |
| RU | Sepsis | 33.597701 | 2007 - 2012 |
| RU | Malaria | 30.421456 | 2007 - 2012 |
| RU | Sepsis | 16.97318 | 2012 - 2017 |
| RU | Malaria | 11.942529 | 2012 - 2017 |
| RW | Sepsis | 2.1685824 | 2007 - 2012 |
| RW | Malaria | 19.195402 | 2007 - 2012 |
| RW | Sepsis | 8.9425287 | 2012 - 2017 |
| RW | Malaria | 46.072797 | 2012 - 2017 |
| SA | Sepsis | 20.003831 | 2007 - 2012 |
| SA | Malaria | 31.83908 | 2007 - 2012 |
| SA | Sepsis | 8.4099617 | 2012 - 2017 |
| SA | Malaria | 10.509579 | 2012 - 2017 |
| SB | Sepsis | 0.5210728 | 2007 - 2012 |
| SB | Malaria | 8.4329502 | 2007 - 2012 |
| SB | Sepsis | 5.2950192 | 2012 - 2017 |
| SB | Malaria | 19.927203 | 2012 - 2017 |
| SC | Sepsis | 0 | 2007 - 2012 |
| SC | Malaria | 3.7318008 | 2007 - 2012 |
| SC | Sepsis | 0 | 2012 - 2017 |
| SC | Malaria | 10.463602 | 2012 - 2017 |
| SD | Sepsis | 5.0076628 | 2007 - 2012 |
| SD | Malaria | 30.206897 | 2007 - 2012 |
| SD | Sepsis | 9.7203065 | 2012 - 2017 |
| SD | Malaria | 53.908046 | 2012 - 2017 |
| SE | Sepsis | 29.655172 | 2007 - 2012 |
| SE | Malaria | 36.084291 | 2007 - 2012 |
| SE | Sepsis | 28.206897 | 2012 - 2017 |
| SE | Malaria | 20.632184 | 2012 - 2017 |
| SG | Sepsis | 21.67433 | 2007 - 2012 |
| SG | Malaria | 42.360153 | 2007 - 2012 |
| SG | Sepsis | 33.272031 | 2012 - 2017 |
| SG | Malaria | 42.996169 | 2012 - 2017 |
| SH | Sepsis | 0 | 2007 - 2012 |
| SH | Malaria | 0.3831418 | 2007 - 2012 |
| SH | Sepsis | 13.770115 | 2012 - 2017 |
| SH | Malaria | 31.747126 | 2012 - 2017 |
| SI | Sepsis | 20.48659 | 2007 - 2012 |
| SI | Malaria | 21.1341 | 2007 - 2012 |
| SI | Sepsis | 23.241379 | 2012 - 2017 |
| SI | Malaria | 17.061303 | 2012 - 2017 |
| SK | Sepsis | 12.02682 | 2007 - 2012 |
| SK | Malaria | 13.195402 | 2007 - 2012 |
| SK | Sepsis | 25.099617 | 2012 - 2017 |
| SK | Malaria | 24.908046 | 2012 - 2017 |
| SL | Sepsis | 0 | 2007 - 2012 |
| SL | Malaria | 4.7318008 | 2007 - 2012 |
| SL | Sepsis | 0 | 2012 - 2017 |
| SL | Malaria | 22.597701 | 2012 - 2017 |
| SN | Sepsis | 1.1992337 | 2007 - 2012 |
| SN | Malaria | 29.750958 | 2007 - 2012 |
| SN | Sepsis | 4.5057471 | 2012 - 2017 |
| SN | Malaria | 41.252874 | 2012 - 2017 |
| SO | Sepsis | 0 | 2007 - 2012 |
| SO | Malaria | 8.1992337 | 2007 - 2012 |
| SO | Sepsis | 6.4559387 | 2012 - 2017 |
| SO | Malaria | 30.498084 | 2012 - 2017 |
| SR | Sepsis | 0.8390805 | 2007 - 2012 |
| SR | Malaria | 8.1340996 | 2007 - 2012 |
| SR | Sepsis | 5.5019157 | 2012 - 2017 |
| SR | Malaria | 16.823755 | 2012 - 2017 |
| SS | Sepsis | 0 | 2012 - 2017 |
| SS | Malaria | 28.835249 | 2012 - 2017 |
| ST | Sepsis | 0 | 2007 - 2012 |
| ST | Malaria | 2.5747126 | 2007 - 2012 |
| ST | Sepsis | 0 | 2012 - 2017 |
| ST | Malaria | 3.9655172 | 2012 - 2017 |
| SV | Sepsis | 9.4099617 | 2007 - 2012 |
| SV | Malaria | 16.076628 | 2007 - 2012 |
| SV | Sepsis | 21.475096 | 2012 - 2017 |
| SV | Malaria | 31.455939 | 2012 - 2017 |
| SY | Sepsis | 7.0689655 | 2007 - 2012 |
| SY | Malaria | 7.4636015 | 2007 - 2012 |
| SY | Sepsis | 31.068966 | 2012 - 2017 |
| SY | Malaria | 23.685824 | 2012 - 2017 |
| SZ | Sepsis | 0 | 2007 - 2012 |
| SZ | Malaria | 5.5632184 | 2007 - 2012 |
| SZ | Sepsis | 0 | 2012 - 2017 |
| SZ | Malaria | 14 | 2012 - 2017 |
| TD | Sepsis | 0 | 2012 - 2017 |
| TD | Malaria | 19.796935 | 2012 - 2017 |
| TG | Sepsis | 0 | 2007 - 2012 |
| TG | Malaria | 11.019157 | 2007 - 2012 |
| TG | Sepsis | 0 | 2012 - 2017 |
| TG | Malaria | 26.915709 | 2012 - 2017 |
| TH | Sepsis | 27.095785 | 2007 - 2012 |
| TH | Malaria | 42.291188 | 2007 - 2012 |
| TH | Sepsis | 46.206897 | 2012 - 2017 |
| TH | Malaria | 42.057471 | 2012 - 2017 |
| TJ | Sepsis | 0.8697318 | 2007 - 2012 |
| TJ | Malaria | 2.8582375 | 2007 - 2012 |
| TJ | Sepsis | 9.3793103 | 2012 - 2017 |
| TJ | Malaria | 9.5478927 | 2012 - 2017 |
| TL | Sepsis | 0 | 2007 - 2012 |
| TL | Malaria | 5.5900383 | 2007 - 2012 |
| TL | Sepsis | 0 | 2012 - 2017 |
| TL | Malaria | 20.701149 | 2012 - 2017 |
| TM | Sepsis | 0 | 2007 - 2012 |
| TM | Malaria | 1.4061303 | 2007 - 2012 |
| TM | Sepsis | 0 | 2012 - 2017 |
| TM | Malaria | 8.7241379 | 2012 - 2017 |
| TN | Sepsis | 6.0076628 | 2007 - 2012 |
| TN | Malaria | 13.961686 | 2007 - 2012 |
| TN | Sepsis | 1.6321839 | 2012 - 2017 |
| TN | Malaria | 2.7394636 | 2012 - 2017 |
| TR | Sepsis | 15.678161 | 2007 - 2012 |
| TR | Malaria | 30.670498 | 2007 - 2012 |
| TR | Sepsis | 13.739464 | 2012 - 2017 |
| TR | Malaria | 25.475096 | 2012 - 2017 |
| TT | Sepsis | 5.0766284 | 2007 - 2012 |
| TT | Malaria | 7.1494253 | 2007 - 2012 |
| TT | Sepsis | 27.735632 | 2012 - 2017 |
| TT | Malaria | 26.022989 | 2012 - 2017 |
| TW | Sepsis | 29.452107 | 2007 - 2012 |
| TW | Malaria | 13.306513 | 2007 - 2012 |
| TW | Sepsis | 54.505747 | 2012 - 2017 |
| TW | Malaria | 16.528736 | 2012 - 2017 |
| TZ | Sepsis | 1.6551724 | 2007 - 2012 |
| TZ | Malaria | 26.739464 | 2007 - 2012 |
| TZ | Sepsis | 7.4252874 | 2012 - 2017 |
| TZ | Malaria | 51.226054 | 2012 - 2017 |
| UA | Sepsis | 20.229885 | 2007 - 2012 |
| UA | Malaria | 23.835249 | 2007 - 2012 |
| UA | Sepsis | 45.153257 | 2012 - 2017 |
| UA | Malaria | 40.302682 | 2012 - 2017 |
| UG | Sepsis | 5.8659004 | 2007 - 2012 |
| UG | Malaria | 40.678161 | 2007 - 2012 |
| UG | Sepsis | 5.348659 | 2012 - 2017 |
| UG | Malaria | 37.536398 | 2012 - 2017 |
| US | Sepsis | 37.038314 | 2007 - 2012 |
| US | Malaria | 38.490421 | 2007 - 2012 |
| US | Sepsis | 30.938697 | 2012 - 2017 |
| US | Malaria | 17.528736 | 2012 - 2017 |
| UY | Sepsis | 8.5172414 | 2007 - 2012 |
| UY | Malaria | 11.463602 | 2007 - 2012 |
| UY | Sepsis | 9.7279693 | 2012 - 2017 |
| UY | Malaria | 12.835249 | 2012 - 2017 |
| UZ | Sepsis | 6.7586207 | 2007 - 2012 |
| UZ | Malaria | 6.1532567 | 2007 - 2012 |
| UZ | Sepsis | 23.306513 | 2012 - 2017 |
| UZ | Malaria | 16.321839 | 2012 - 2017 |
| VC | Sepsis | 0 | 2007 - 2012 |
| VC | Malaria | 2.5938697 | 2007 - 2012 |
| VC | Sepsis | 0 | 2012 - 2017 |
| VC | Malaria | 11.019157 | 2012 - 2017 |
| VE | Sepsis | 8.3256705 | 2007 - 2012 |
| VE | Malaria | 23.022989 | 2007 - 2012 |
| VE | Sepsis | 6.1455939 | 2012 - 2017 |
| VE | Malaria | 17.785441 | 2012 - 2017 |
| VN | Sepsis | 14.747126 | 2007 - 2012 |
| VN | Malaria | 29.881226 | 2007 - 2012 |
| VN | Sepsis | 23.923372 | 2012 - 2017 |
| VN | Malaria | 50.563218 | 2012 - 2017 |
| VU | Sepsis | 0 | 2007 - 2012 |
| VU | Malaria | 6.2183908 | 2007 - 2012 |
| VU | Sepsis | 0 | 2012 - 2017 |
| VU | Malaria | 8.0766284 | 2012 - 2017 |
| YE | Sepsis | 2.0727969 | 2007 - 2012 |
| YE | Malaria | 21.45977 | 2007 - 2012 |
| YE | Sepsis | 4.8582375 | 2012 - 2017 |
| YE | Malaria | 26.153257 | 2012 - 2017 |
| YT | Sepsis | 0 | 2007 - 2012 |
| YT | Malaria | 1.0114943 | 2007 - 2012 |
| YT | Sepsis | 0 | 2012 - 2017 |
| YT | Malaria | 3.1685824 | 2012 - 2017 |
| ZA | Sepsis | 6.6398467 | 2007 - 2012 |
| ZA | Malaria | 47.471264 | 2007 - 2012 |
| ZA | Sepsis | 9.2183908 | 2012 - 2017 |
| ZA | Malaria | 38.885057 | 2012 - 2017 |
| ZM | Sepsis | 1.6360153 | 2007 - 2012 |
| ZM | Malaria | 19.823755 | 2007 - 2012 |
| ZM | Sepsis | 10.800766 | 2012 - 2017 |
| ZM | Malaria | 50.157088 | 2012 - 2017 |
| ZW | Sepsis | 1.6475096 | 2007 - 2012 |
| ZW | Malaria | 11.996169 | 2007 - 2012 |
| ZW | Sepsis | 13.636015 | 2012 - 2017 |
| ZW | Malaria | 48.003831 | 2012 - 2017 |

2007 - 2012, 6/24/2007 to 6/23/2012; 2012 - 2017, 6/24/2012 to 6/24/2017; ISO: International Organization for Standardization 3166-1 alpha-2 codes as follows:

AD, Andorra; AE, United Arab Emirates; AF, Afghanistan; AG, Antigua and Barbuda; AL, Albania; AM, Armenia; AO, Angola; AR, Argentina; AS, American Samoa; AT, Austria; AU, Australia; AZ, Azerbaijan; BA, Bosnia and Herzegovina; BB, Barbados; BD, Bangladesh; BE, Belgium; BF, Burkina Faso; BG, Bulgaria; BH, Bahrain; BI, Burundi; BJ, Benin; BN, Brunei Darussalam; BO, Bolivia; BR, Brazil; BS, Bahamas; BT, Bhutan; BW, Botswana; BY, Belarus; BZ, Belize; CA, Canada; CC, Cocos (Keeling) Islands; CF, Central African Republic; CG, Congo; CH, Switzerland; CI, Côte d'Ivoire; CL, Chile; CM, Cameroon; CN, China; CO, Colombia; CR, Costa Rica; CU, Cuba; CV, Cabo Verde; CW, Curaçao; CY, Cyprus; CZ, Czechia; DE, Germany; DJ, Djibouti; DK, Denmark; DM, Dominica; DO, Dominican Republic; DZ, Algeria; EC, Ecuador; EE, Estonia; EG, Egypt; ER, Eritrea; ES, Spain; ET, Ethiopia; FI, Finland; FJ, Fiji; FR, France; GA, Gabon; GB, United Kingdom of Great Britain and Northern Ireland; GD, Grenada; GE, Georgia; GF, French Guiana; GG, Guernsey; GH, Ghana; GI, Gibraltar; GM, Gambia; GN, Guinea; GP, Guadeloupe; GQ, Equatorial Guinea; GR, Greece; GT, Guatemala; GU, Guam; GW, Guinea-Bissau; GY, Guyana; HK, Hong Kong; HN, Honduras; HR, Croatia; HT, Haiti; HU, Hungary; ID, Indonesia; IE, Ireland; IL, Israel; IM, Isle of Man; IN, India; IQ, Iraq; IR, Iran; IS, Iceland; IT, Italy; JE, Jersey; JM, Jamaica; JO, Jordan; JP, Japan; KE, Kenya; KG, Kyrgyzstan; KH, Cambodia; KR, Korea; KW, Kuwait; KY, Cayman Islands; KZ, Kazakhstan; LA, Lao People's Democratic Republic; LB, Lebanon; LC, Saint Lucia; LK, Sri Lanka; LR, Liberia; LS, Lesotho; LT, Lithuania; LU, Luxembourg; LV, Latvia; LY, Libya; MA, Morocco; MD, Moldova; ME, Montenegro; MG, Madagascar; MK, Macedonia; ML, Mali; MM, Myanmar; MN, Mongolia; MO, Macao; MQ, Martinique; MR, Mauritania; MT, Malta; MU, Mauritius; MV, Maldives; MW, Malawi; MX, Mexico; MY, Malaysia; MZ, Mozambique; NA, Namibia; NC, New Caledonia; NE, Niger; NG, Nigeria; NI, Nicaragua; NL, Netherlands; NO, Norway; NP, Nepal; NZ, New Zealand; OM, Oman; PA, Panama; PE, Peru; PF, French Polynesia; PG, Papua New Guinea; PH, Philippines; PK, Pakistan; PL, Poland; PR, Puerto Rico; PS, Palestine; PT, Portugal; PY, Paraguay; QA, Qatar; RE, Réunion; RO, Romania; RS, Serbia; RU, Russian Federation; RW, Rwanda; SA, Saudi Arabia; SB, Solomon Islands; SC, Seychelles; SD, Sudan; SE, Sweden; SG, Singapore; SH, Saint Helena; SI, Slovenia; SK, Slovakia; SL, Sierra Leone; SN, Senegal; SO, Somalia; SR, Suriname; SS, South Sudan; ST, Sao Tome and Principe; SV, El Salvador; SY, Syrian Arab Republic; SZ, Swaziland; TD, Chad; TG, Togo; TH, Thailand; TJ, Tajikistan; TL, Timor-Leste; TM, Turkmenistan; TN, Tunisia; TR, Turkey; TT, Trinidad and Tobago; TW, Taiwan; TZ, United Republic of Tanzania; UA, Ukraine; UG, Uganda; US, United States of America; UY, Uruguay; UZ, Uzbekistan; VC, Saint Vincent and the Grenadines; VE, Venezuela; VN, Viet Nam; VU, Vanuatu; YE, Yemen; YT, Mayotte; ZA, South Africa; ZM, Zambia; ZW, Zimbabwe

**Table S6. Influenza, Myocardial Infarction, Sepsis, and Stroke Relative Search Volume Time Series Dataset**

| **Week Start** | **Sepsis** | **Stroke** | **Myocardial Infarction** | **Influenza** | **Period** | **Year** | **Week** |
| --- | --- | --- | --- | --- | --- | --- | --- |
| 6/24/2012 | 3 | 16 | 9 | 15 | 1 | 2012 | 26 |
| 7/1/2012 | 3 | 17 | 8 | 15 | 2 | 2012 | 27 |
| 7/8/2012 | 4 | 16 | 9 | 15 | 3 | 2012 | 28 |
| 7/15/2012 | 4 | 16 | 9 | 15 | 4 | 2012 | 29 |
| 7/22/2012 | 3 | 16 | 9 | 14 | 5 | 2012 | 30 |
| 7/29/2012 | 3 | 16 | 8 | 14 | 6 | 2012 | 31 |
| 8/5/2012 | 3 | 15 | 8 | 14 | 7 | 2012 | 32 |
| 8/12/2012 | 3 | 15 | 9 | 14 | 8 | 2012 | 33 |
| 8/19/2012 | 4 | 16 | 10 | 14 | 9 | 2012 | 34 |
| 8/26/2012 | 4 | 16 | 9 | 16 | 10 | 2012 | 35 |
| 9/2/2012 | 4 | 15 | 16 | 17 | 11 | 2012 | 36 |
| 9/9/2012 | 4 | 16 | 11 | 20 | 12 | 2012 | 37 |
| 9/16/2012 | 4 | 15 | 10 | 22 | 13 | 2012 | 38 |
| 9/23/2012 | 4 | 16 | 10 | 26 | 14 | 2012 | 39 |
| 9/30/2012 | 4 | 16 | 10 | 28 | 15 | 2012 | 40 |
| 10/7/2012 | 4 | 15 | 9 | 29 | 16 | 2012 | 41 |
| 10/14/2012 | 4 | 16 | 9 | 30 | 17 | 2012 | 42 |
| 10/21/2012 | 4 | 16 | 9 | 30 | 18 | 2012 | 43 |
| 10/28/2012 | 3 | 16 | 9 | 28 | 19 | 2012 | 44 |
| 11/4/2012 | 4 | 15 | 9 | 29 | 20 | 2012 | 45 |
| 11/11/2012 | 5 | 15 | 10 | 30 | 21 | 2012 | 46 |
| 11/18/2012 | 4 | 15 | 9 | 27 | 22 | 2012 | 47 |
| 11/25/2012 | 4 | 16 | 9 | 30 | 23 | 2012 | 48 |
| 12/2/2012 | 4 | 16 | 9 | 34 | 24 | 2012 | 49 |
| 12/9/2012 | 3 | 15 | 9 | 33 | 25 | 2012 | 50 |
| 12/16/2012 | 3 | 14 | 8 | 36 | 26 | 2012 | 51 |
| 12/23/2012 | 3 | 13 | 8 | 43 | 27 | 2012 | 52 |
| 12/30/2012 | 4 | 14 | 9 | 52 | 28 | 2013 | 1 |
| 1/6/2013 | 4 | 17 | 9 | 100 | 29 | 2013 | 2 |
| 1/13/2013 | 4 | 16 | 9 | 90 | 30 | 2013 | 3 |
| 1/20/2013 | 4 | 16 | 9 | 71 | 31 | 2013 | 4 |
| 1/27/2013 | 4 | 16 | 10 | 64 | 32 | 2013 | 5 |
| 2/3/2013 | 4 | 16 | 10 | 56 | 33 | 2013 | 6 |
| 2/10/2013 | 4 | 16 | 10 | 48 | 34 | 2013 | 7 |
| 2/17/2013 | 4 | 16 | 10 | 46 | 35 | 2013 | 8 |
| 2/24/2013 | 4 | 16 | 10 | 39 | 36 | 2013 | 9 |
| 3/3/2013 | 4 | 16 | 10 | 35 | 37 | 2013 | 10 |
| 3/10/2013 | 4 | 16 | 10 | 31 | 38 | 2013 | 11 |
| 3/17/2013 | 4 | 17 | 10 | 28 | 39 | 2013 | 12 |
| 3/24/2013 | 4 | 16 | 10 | 25 | 40 | 2013 | 13 |
| 3/31/2013 | 4 | 16 | 10 | 31 | 41 | 2013 | 14 |
| 4/7/2013 | 4 | 18 | 10 | 31 | 42 | 2013 | 15 |
| 4/14/2013 | 4 | 16 | 10 | 30 | 43 | 2013 | 16 |
| 4/21/2013 | 4 | 16 | 10 | 27 | 44 | 2013 | 17 |
| 4/28/2013 | 4 | 16 | 10 | 23 | 45 | 2013 | 18 |
| 5/5/2013 | 4 | 16 | 12 | 21 | 46 | 2013 | 19 |
| 5/12/2013 | 4 | 16 | 10 | 19 | 47 | 2013 | 20 |
| 5/19/2013 | 4 | 16 | 10 | 19 | 48 | 2013 | 21 |
| 5/26/2013 | 4 | 16 | 9 | 19 | 49 | 2013 | 22 |
| 6/2/2013 | 4 | 16 | 9 | 17 | 50 | 2013 | 23 |
| 6/9/2013 | 4 | 16 | 9 | 18 | 51 | 2013 | 24 |
| 6/16/2013 | 4 | 15 | 10 | 15 | 52 | 2013 | 25 |
| 6/23/2013 | 4 | 16 | 10 | 15 | 53 | 2013 | 26 |
| 6/30/2013 | 3 | 16 | 9 | 15 | 54 | 2013 | 27 |
| 7/7/2013 | 4 | 17 | 9 | 14 | 55 | 2013 | 28 |
| 7/14/2013 | 4 | 18 | 9 | 15 | 56 | 2013 | 29 |
| 7/21/2013 | 4 | 15 | 9 | 14 | 57 | 2013 | 30 |
| 7/28/2013 | 4 | 15 | 9 | 13 | 58 | 2013 | 31 |
| 8/4/2013 | 4 | 15 | 9 | 14 | 59 | 2013 | 32 |
| 8/11/2013 | 4 | 15 | 9 | 16 | 60 | 2013 | 33 |
| 8/18/2013 | 4 | 15 | 9 | 16 | 61 | 2013 | 34 |
| 8/25/2013 | 4 | 15 | 9 | 15 | 62 | 2013 | 35 |
| 9/1/2013 | 4 | 16 | 9 | 17 | 63 | 2013 | 36 |
| 9/8/2013 | 5 | 15 | 9 | 20 | 64 | 2013 | 37 |
| 9/15/2013 | 4 | 15 | 9 | 23 | 65 | 2013 | 38 |
| 9/22/2013 | 4 | 16 | 10 | 27 | 66 | 2013 | 39 |
| 9/29/2013 | 4 | 15 | 9 | 30 | 67 | 2013 | 40 |
| 10/6/2013 | 4 | 16 | 9 | 32 | 68 | 2013 | 41 |
| 10/13/2013 | 4 | 16 | 9 | 32 | 69 | 2013 | 42 |
| 10/20/2013 | 4 | 16 | 10 | 33 | 70 | 2013 | 43 |
| 10/27/2013 | 4 | 18 | 9 | 31 | 71 | 2013 | 44 |
| 11/3/2013 | 4 | 17 | 10 | 32 | 72 | 2013 | 45 |
| 11/10/2013 | 4 | 16 | 10 | 32 | 73 | 2013 | 46 |
| 11/17/2013 | 4 | 16 | 10 | 30 | 74 | 2013 | 47 |
| 11/24/2013 | 4 | 16 | 9 | 28 | 75 | 2013 | 48 |
| 12/1/2013 | 4 | 16 | 9 | 29 | 76 | 2013 | 49 |
| 12/8/2013 | 4 | 15 | 9 | 29 | 77 | 2013 | 50 |
| 12/15/2013 | 3 | 14 | 9 | 31 | 78 | 2013 | 51 |
| 12/22/2013 | 3 | 14 | 9 | 36 | 79 | 2013 | 52 |
| 12/29/2013 | 3 | 14 | 9 | 45 | 80 | 2014 | 1 |
| 1/5/2014 | 4 | 16 | 10 | 59 | 81 | 2014 | 2 |
| 1/12/2014 | 4 | 16 | 10 | 57 | 82 | 2014 | 3 |
| 1/19/2014 | 4 | 16 | 10 | 59 | 83 | 2014 | 4 |
| 1/26/2014 | 4 | 16 | 11 | 59 | 84 | 2014 | 5 |
| 2/2/2014 | 4 | 17 | 11 | 51 | 85 | 2014 | 6 |
| 2/9/2014 | 4 | 16 | 11 | 45 | 86 | 2014 | 7 |
| 2/16/2014 | 4 | 16 | 11 | 43 | 87 | 2014 | 8 |
| 2/23/2014 | 4 | 16 | 11 | 39 | 88 | 2014 | 9 |
| 3/2/2014 | 4 | 16 | 10 | 35 | 89 | 2014 | 10 |
| 3/9/2014 | 4 | 16 | 11 | 32 | 90 | 2014 | 11 |
| 3/16/2014 | 4 | 16 | 10 | 30 | 91 | 2014 | 12 |
| 3/23/2014 | 4 | 16 | 10 | 28 | 92 | 2014 | 13 |
| 3/30/2014 | 4 | 16 | 10 | 25 | 93 | 2014 | 14 |
| 4/6/2014 | 4 | 16 | 10 | 23 | 94 | 2014 | 15 |
| 4/13/2014 | 4 | 16 | 10 | 23 | 95 | 2014 | 16 |
| 4/20/2014 | 4 | 17 | 10 | 24 | 96 | 2014 | 17 |
| 4/27/2014 | 4 | 17 | 10 | 21 | 97 | 2014 | 18 |
| 5/4/2014 | 4 | 17 | 10 | 21 | 98 | 2014 | 19 |
| 5/11/2014 | 4 | 17 | 10 | 18 | 99 | 2014 | 20 |
| 5/18/2014 | 4 | 17 | 9 | 17 | 100 | 2014 | 21 |
| 5/25/2014 | 4 | 16 | 9 | 16 | 101 | 2014 | 22 |
| 6/1/2014 | 4 | 16 | 9 | 16 | 102 | 2014 | 23 |
| 6/8/2014 | 4 | 16 | 9 | 14 | 103 | 2014 | 24 |
| 6/15/2014 | 4 | 16 | 9 | 14 | 104 | 2014 | 25 |
| 6/22/2014 | 4 | 16 | 9 | 13 | 105 | 2014 | 26 |
| 6/29/2014 | 4 | 16 | 9 | 15 | 106 | 2014 | 27 |
| 7/6/2014 | 4 | 16 | 9 | 14 | 107 | 2014 | 28 |
| 7/13/2014 | 4 | 16 | 9 | 13 | 108 | 2014 | 29 |
| 7/20/2014 | 4 | 17 | 9 | 13 | 109 | 2014 | 30 |
| 7/27/2014 | 4 | 16 | 9 | 14 | 110 | 2014 | 31 |
| 8/3/2014 | 4 | 16 | 8 | 15 | 111 | 2014 | 32 |
| 8/10/2014 | 4 | 16 | 9 | 16 | 112 | 2014 | 33 |
| 8/17/2014 | 4 | 16 | 9 | 16 | 113 | 2014 | 34 |
| 8/24/2014 | 4 | 16 | 9 | 17 | 114 | 2014 | 35 |
| 8/31/2014 | 4 | 17 | 9 | 17 | 115 | 2014 | 36 |
| 9/7/2014 | 5 | 16 | 9 | 21 | 116 | 2014 | 37 |
| 9/14/2014 | 5 | 16 | 9 | 24 | 117 | 2014 | 38 |
| 9/21/2014 | 4 | 16 | 9 | 27 | 118 | 2014 | 39 |
| 9/28/2014 | 4 | 17 | 9 | 31 | 119 | 2014 | 40 |
| 10/5/2014 | 4 | 16 | 9 | 38 | 120 | 2014 | 41 |
| 10/12/2014 | 4 | 16 | 9 | 41 | 121 | 2014 | 42 |
| 10/19/2014 | 4 | 16 | 9 | 39 | 122 | 2014 | 43 |
| 10/26/2014 | 4 | 19 | 10 | 34 | 123 | 2014 | 44 |
| 11/2/2014 | 4 | 17 | 9 | 34 | 124 | 2014 | 45 |
| 11/9/2014 | 5 | 17 | 10 | 33 | 125 | 2014 | 46 |
| 11/16/2014 | 4 | 17 | 10 | 34 | 126 | 2014 | 47 |
| 11/23/2014 | 4 | 16 | 9 | 32 | 127 | 2014 | 48 |
| 11/30/2014 | 5 | 16 | 10 | 39 | 128 | 2014 | 49 |
| 12/7/2014 | 4 | 16 | 10 | 41 | 129 | 2014 | 50 |
| 12/14/2014 | 4 | 15 | 9 | 49 | 130 | 2014 | 51 |
| 12/21/2014 | 3 | 14 | 8 | 56 | 131 | 2014 | 52 |
| 12/28/2014 | 4 | 14 | 9 | 63 | 132 | 2015 | 1 |
| 1/4/2015 | 4 | 16 | 10 | 65 | 133 | 2015 | 2 |
| 1/11/2015 | 5 | 16 | 10 | 60 | 134 | 2015 | 3 |
| 1/18/2015 | 4 | 16 | 10 | 62 | 135 | 2015 | 4 |
| 1/25/2015 | 4 | 16 | 10 | 58 | 136 | 2015 | 5 |
| 2/1/2015 | 4 | 17 | 10 | 58 | 137 | 2015 | 6 |
| 2/8/2015 | 5 | 16 | 10 | 56 | 138 | 2015 | 7 |
| 2/15/2015 | 4 | 17 | 10 | 66 | 139 | 2015 | 8 |
| 2/22/2015 | 4 | 17 | 10 | 59 | 140 | 2015 | 9 |
| 3/1/2015 | 4 | 17 | 10 | 52 | 141 | 2015 | 10 |
| 3/8/2015 | 4 | 17 | 10 | 41 | 142 | 2015 | 11 |
| 3/15/2015 | 4 | 17 | 10 | 35 | 143 | 2015 | 12 |
| 3/22/2015 | 4 | 17 | 10 | 30 | 144 | 2015 | 13 |
| 3/29/2015 | 4 | 16 | 10 | 27 | 145 | 2015 | 14 |
| 4/5/2015 | 4 | 17 | 10 | 28 | 146 | 2015 | 15 |
| 4/12/2015 | 4 | 17 | 10 | 27 | 147 | 2015 | 16 |
| 4/19/2015 | 4 | 18 | 10 | 25 | 148 | 2015 | 17 |
| 4/26/2015 | 4 | 17 | 10 | 22 | 149 | 2015 | 18 |
| 5/3/2015 | 5 | 17 | 10 | 23 | 150 | 2015 | 19 |
| 5/10/2015 | 4 | 18 | 9 | 22 | 151 | 2015 | 20 |
| 5/17/2015 | 4 | 17 | 9 | 22 | 152 | 2015 | 21 |
| 5/24/2015 | 4 | 17 | 9 | 20 | 153 | 2015 | 22 |
| 5/31/2015 | 4 | 18 | 10 | 19 | 154 | 2015 | 23 |
| 6/7/2015 | 4 | 19 | 10 | 18 | 155 | 2015 | 24 |
| 6/14/2015 | 4 | 18 | 9 | 17 | 156 | 2015 | 25 |
| 6/21/2015 | 4 | 18 | 9 | 16 | 157 | 2015 | 26 |
| 6/28/2015 | 4 | 18 | 9 | 15 | 158 | 2015 | 27 |
| 7/5/2015 | 4 | 17 | 9 | 15 | 159 | 2015 | 28 |
| 7/12/2015 | 4 | 17 | 9 | 16 | 160 | 2015 | 29 |
| 7/19/2015 | 4 | 17 | 9 | 15 | 161 | 2015 | 30 |
| 7/26/2015 | 4 | 17 | 9 | 14 | 162 | 2015 | 31 |
| 8/2/2015 | 4 | 17 | 9 | 14 | 163 | 2015 | 32 |
| 8/9/2015 | 4 | 18 | 9 | 15 | 164 | 2015 | 33 |
| 8/16/2015 | 4 | 17 | 9 | 16 | 165 | 2015 | 34 |
| 8/23/2015 | 4 | 16 | 9 | 17 | 166 | 2015 | 35 |
| 8/30/2015 | 4 | 16 | 9 | 18 | 167 | 2015 | 36 |
| 9/6/2015 | 5 | 17 | 9 | 20 | 168 | 2015 | 37 |
| 9/13/2015 | 4 | 17 | 10 | 24 | 169 | 2015 | 38 |
| 9/20/2015 | 5 | 18 | 10 | 26 | 170 | 2015 | 39 |
| 9/27/2015 | 4 | 18 | 10 | 31 | 171 | 2015 | 40 |
| 10/4/2015 | 5 | 18 | 10 | 37 | 172 | 2015 | 41 |
| 10/11/2015 | 4 | 18 | 10 | 38 | 173 | 2015 | 42 |
| 10/18/2015 | 4 | 18 | 10 | 39 | 174 | 2015 | 43 |
| 10/25/2015 | 4 | 19 | 10 | 37 | 175 | 2015 | 44 |
| 11/1/2015 | 5 | 18 | 10 | 36 | 176 | 2015 | 45 |
| 11/8/2015 | 5 | 18 | 10 | 34 | 177 | 2015 | 46 |
| 11/15/2015 | 5 | 18 | 10 | 33 | 178 | 2015 | 47 |
| 11/22/2015 | 4 | 17 | 12 | 30 | 179 | 2015 | 48 |
| 11/29/2015 | 4 | 17 | 11 | 31 | 180 | 2015 | 49 |
| 12/6/2015 | 4 | 17 | 10 | 35 | 181 | 2015 | 50 |
| 12/13/2015 | 4 | 17 | 10 | 31 | 182 | 2015 | 51 |
| 12/20/2015 | 4 | 16 | 9 | 30 | 183 | 2015 | 52 |
| 12/27/2015 | 4 | 16 | 10 | 31 | 184 | 2015 | 53 |
| 1/3/2016 | 5 | 17 | 10 | 32 | 185 | 2016 | 1 |
| 1/10/2016 | 4 | 17 | 11 | 38 | 186 | 2016 | 2 |
| 1/17/2016 | 4 | 17 | 11 | 53 | 187 | 2016 | 3 |
| 1/24/2016 | 7 | 17 | 11 | 70 | 188 | 2016 | 4 |
| 1/31/2016 | 5 | 19 | 10 | 70 | 189 | 2016 | 5 |
| 2/7/2016 | 5 | 18 | 11 | 68 | 190 | 2016 | 6 |
| 2/14/2016 | 5 | 18 | 11 | 69 | 191 | 2016 | 7 |
| 2/21/2016 | 5 | 18 | 11 | 67 | 192 | 2016 | 8 |
| 2/28/2016 | 5 | 18 | 12 | 63 | 193 | 2016 | 9 |
| 3/6/2016 | 5 | 18 | 11 | 61 | 194 | 2016 | 10 |
| 3/13/2016 | 5 | 18 | 11 | 51 | 195 | 2016 | 11 |
| 3/20/2016 | 5 | 18 | 10 | 45 | 196 | 2016 | 12 |
| 3/27/2016 | 8 | 20 | 12 | 43 | 197 | 2016 | 13 |
| 4/3/2016 | 5 | 18 | 11 | 38 | 198 | 2016 | 14 |
| 4/10/2016 | 5 | 18 | 11 | 33 | 199 | 2016 | 15 |
| 4/17/2016 | 5 | 19 | 10 | 30 | 200 | 2016 | 16 |
| 4/24/2016 | 5 | 19 | 10 | 29 | 201 | 2016 | 17 |
| 5/1/2016 | 5 | 18 | 10 | 25 | 202 | 2016 | 18 |
| 5/8/2016 | 5 | 19 | 10 | 23 | 203 | 2016 | 19 |
| 5/15/2016 | 5 | 19 | 10 | 23 | 204 | 2016 | 20 |
| 5/22/2016 | 5 | 19 | 10 | 25 | 205 | 2016 | 21 |
| 5/29/2016 | 6 | 19 | 10 | 22 | Period | 2016 | 22 |
| 6/5/2016 | 8 | 19 | 10 | 20 | 207 | 2016 | 23 |
| 6/12/2016 | 5 | 18 | 10 | 19 | 208 | 2016 | 24 |
| 6/19/2016 | 5 | 18 | 9 | 17 | 209 | 2016 | 25 |
| 6/26/2016 | 5 | 18 | 9 | 17 | 210 | 2016 | 26 |
| 7/3/2016 | 5 | 18 | 9 | 15 | 211 | 2016 | 27 |
| 7/10/2016 | 5 | 18 | 9 | 16 | 212 | 2016 | 28 |
| 7/17/2016 | 5 | 19 | 9 | 18 | 213 | 2016 | 29 |
| 7/24/2016 | 5 | 18 | 9 | 15 | 214 | 2016 | 30 |
| 7/31/2016 | 4 | 18 | 9 | 14 | 215 | 2016 | 31 |
| 8/7/2016 | 4 | 18 | 9 | 14 | 216 | 2016 | 32 |
| 8/14/2016 | 4 | 17 | 9 | 15 | 217 | 2016 | 33 |
| 8/21/2016 | 5 | 17 | 9 | 17 | 218 | 2016 | 34 |
| 8/28/2016 | 5 | 18 | 9 | 18 | 219 | 2016 | 35 |
| 9/4/2016 | 5 | 18 | 10 | 21 | 220 | 2016 | 36 |
| 9/11/2016 | 7 | 19 | 10 | 26 | 221 | 2016 | 37 |
| 9/18/2016 | 6 | 18 | 10 | 29 | 222 | 2016 | 38 |
| 9/25/2016 | 6 | 18 | 10 | 34 | 223 | 2016 | 39 |
| 10/2/2016 | 6 | 19 | 11 | 36 | 224 | 2016 | 40 |
| 10/9/2016 | 5 | 19 | 10 | 38 | 225 | 2016 | 41 |
| 10/16/2016 | 6 | 20 | 10 | 38 | 226 | 2016 | 42 |
| 10/23/2016 | 5 | 20 | 10 | 39 | 227 | 2016 | 43 |
| 10/30/2016 | 5 | 21 | 11 | 37 | 228 | 2016 | 44 |
| 11/6/2016 | 5 | 19 | 11 | 38 | 229 | 2016 | 45 |
| 11/13/2016 | 5 | 19 | 10 | 40 | 230 | 2016 | 46 |
| 11/20/2016 | 5 | 18 | 10 | 36 | 231 | 2016 | 47 |
| 11/27/2016 | 5 | 19 | 10 | 41 | 232 | 2016 | 48 |
| 12/4/2016 | 5 | 18 | 10 | 41 | 233 | 2016 | 49 |
| 12/11/2016 | 5 | 18 | 10 | 44 | 234 | 2016 | 50 |
| 12/18/2016 | 5 | 17 | 12 | 50 | 235 | 2016 | 51 |
| 12/25/2016 | 5 | 18 | 13 | 57 | 236 | 2016 | 52 |
| 1/1/2017 | 6 | 18 | 11 | 61 | 237 | 2017 | 1 |
| 1/8/2017 | 5 | 19 | 11 | 61 | 238 | 2017 | 2 |
| 1/15/2017 | 5 | 19 | 11 | 61 | 239 | 2017 | 3 |
| 1/22/2017 | 6 | 21 | 11 | 65 | 240 | 2017 | 4 |
| 1/29/2017 | 6 | 20 | 11 | 67 | 241 | 2017 | 5 |
| 2/5/2017 | 6 | 20 | 11 | 65 | 242 | 2017 | 6 |
| 2/12/2017 | 5 | 20 | 11 | 58 | 243 | 2017 | 7 |
| 2/19/2017 | 6 | 20 | 11 | 52 | 244 | 2017 | 8 |
| 2/26/2017 | 6 | 20 | 12 | 43 | 245 | 2017 | 9 |
| 3/5/2017 | 6 | 20 | 11 | 38 | 246 | 2017 | 10 |
| 3/12/2017 | 6 | 20 | 11 | 36 | 247 | 2017 | 11 |
| 3/19/2017 | 6 | 21 | 11 | 35 | 248 | 2017 | 12 |
| 3/26/2017 | 7 | 22 | 11 | 32 | 249 | 2017 | 13 |
| 4/2/2017 | 6 | 21 | 11 | 29 | 250 | 2017 | 14 |
| 4/9/2017 | 5 | 21 | 10 | 26 | 251 | 2017 | 15 |
| 4/16/2017 | 5 | 21 | 11 | 27 | 252 | 2017 | 16 |
| 4/23/2017 | 6 | 21 | 11 | 25 | 253 | 2017 | 17 |
| 4/30/2017 | 5 | 22 | 10 | 23 | 254 | 2017 | 18 |
| 5/7/2017 | 5 | 22 | 11 | 24 | 255 | 2017 | 19 |
| 5/14/2017 | 5 | 22 | 10 | 22 | 256 | 2017 | 20 |
| 5/21/2017 | 5 | 22 | 10 | 22 | 257 | 2017 | 21 |
| 5/28/2017 | 6 | 21 | 10 | 21 | 258 | 2017 | 22 |
| 6/4/2017 | 5 | 20 | 10 | 21 | 259 | 2017 | 23 |
| 6/11/2017 | 5 | 21 | 10 | 19 | 260 | 2017 | 24 |
| 6/18/2017 | 5 | 23 | 10 | 19 | 261 | 2017 | 25 |

**Table S7. Sepsis (Topic) Relative Search Volume Time Series Dataset for the United States**

| **Week Start** | **Sepsis: (1/1/2012 – 12/31/2016)** | **Year** | **Week** | **Period** |
| --- | --- | --- | --- | --- |
| 1/1/2012 | 20 | 2012 | 1 | 1 |
| 1/8/2012 | 24 | 2012 | 2 | 2 |
| 1/15/2012 | 23 | 2012 | 3 | 3 |
| 1/22/2012 | 23 | 2012 | 4 | 4 |
| 1/29/2012 | 24 | 2012 | 5 | 5 |
| 2/5/2012 | 25 | 2012 | 6 | 6 |
| 2/12/2012 | 26 | 2012 | 7 | 7 |
| 2/19/2012 | 24 | 2012 | 8 | 8 |
| 2/26/2012 | 25 | 2012 | 9 | 9 |
| 3/4/2012 | 28 | 2012 | 10 | 10 |
| 3/11/2012 | 24 | 2012 | 11 | 11 |
| 3/18/2012 | 26 | 2012 | 12 | 12 |
| 3/25/2012 | 24 | 2012 | 13 | 13 |
| 4/1/2012 | 25 | 2012 | 14 | 14 |
| 4/8/2012 | 24 | 2012 | 15 | 15 |
| 4/15/2012 | 24 | 2012 | 16 | 16 |
| 4/22/2012 | 24 | 2012 | 17 | 17 |
| 4/29/2012 | 23 | 2012 | 18 | 18 |
| 5/6/2012 | 23 | 2012 | 19 | 19 |
| 5/13/2012 | 27 | 2012 | 20 | 20 |
| 5/20/2012 | 22 | 2012 | 21 | 21 |
| 5/27/2012 | 25 | 2012 | 22 | 22 |
| 6/3/2012 | 23 | 2012 | 23 | 23 |
| 6/10/2012 | 23 | 2012 | 24 | 24 |
| 6/17/2012 | 23 | 2012 | 25 | 25 |
| 6/24/2012 | 22 | 2012 | 26 | 26 |
| 7/1/2012 | 22 | 2012 | 27 | 27 |
| 7/8/2012 | 39 | 2012 | 28 | 28 |
| 7/15/2012 | 33 | 2012 | 29 | 29 |
| 7/22/2012 | 25 | 2012 | 30 | 30 |
| 7/29/2012 | 23 | 2012 | 31 | 31 |
| 8/5/2012 | 24 | 2012 | 32 | 32 |
| 8/12/2012 | 23 | 2012 | 33 | 33 |
| 8/19/2012 | 23 | 2012 | 34 | 34 |
| 8/26/2012 | 23 | 2012 | 35 | 35 |
| 9/2/2012 | 24 | 2012 | 36 | 36 |
| 9/9/2012 | 26 | 2012 | 37 | 37 |
| 9/16/2012 | 25 | 2012 | 38 | 38 |
| 9/23/2012 | 26 | 2012 | 39 | 39 |
| 9/30/2012 | 27 | 2012 | 40 | 40 |
| 10/7/2012 | 25 | 2012 | 41 | 41 |
| 10/14/2012 | 27 | 2012 | 42 | 42 |
| 10/21/2012 | 25 | 2012 | 43 | 43 |
| 10/28/2012 | 23 | 2012 | 44 | 44 |
| 11/4/2012 | 23 | 2012 | 45 | 45 |
| 11/11/2012 | 33 | 2012 | 46 | 46 |
| 11/18/2012 | 23 | 2012 | 47 | 47 |
| 11/25/2012 | 24 | 2012 | 48 | 48 |
| 12/2/2012 | 24 | 2012 | 49 | 49 |
| 12/9/2012 | 23 | 2012 | 50 | 50 |
| 12/16/2012 | 22 | 2012 | 51 | 51 |
| 12/23/2012 | 20 | 2012 | 52 | 52 |
| 12/30/2012 | 24 | 2013 | 1 | 53 |
| 1/6/2013 | 26 | 2013 | 2 | 54 |
| 1/13/2013 | 23 | 2013 | 3 | 55 |
| 1/20/2013 | 25 | 2013 | 4 | 56 |
| 1/27/2013 | 26 | 2013 | 5 | 57 |
| 2/3/2013 | 27 | 2013 | 6 | 58 |
| 2/10/2013 | 28 | 2013 | 7 | 59 |
| 2/17/2013 | 28 | 2013 | 8 | 60 |
| 2/24/2013 | 27 | 2013 | 9 | 61 |
| 3/3/2013 | 27 | 2013 | 10 | 62 |
| 3/10/2013 | 25 | 2013 | 11 | 63 |
| 3/17/2013 | 27 | 2013 | 12 | 64 |
| 3/24/2013 | 27 | 2013 | 13 | 65 |
| 3/31/2013 | 26 | 2013 | 14 | 66 |
| 4/7/2013 | 27 | 2013 | 15 | 67 |
| 4/14/2013 | 32 | 2013 | 16 | 68 |
| 4/21/2013 | 32 | 2013 | 17 | 69 |
| 4/28/2013 | 26 | 2013 | 18 | 70 |
| 5/5/2013 | 25 | 2013 | 19 | 71 |
| 5/12/2013 | 24 | 2013 | 20 | 72 |
| 5/19/2013 | 22 | 2013 | 21 | 73 |
| 5/26/2013 | 24 | 2013 | 22 | 74 |
| 6/2/2013 | 24 | 2013 | 23 | 75 |
| 6/9/2013 | 26 | 2013 | 24 | 76 |
| 6/16/2013 | 25 | 2013 | 25 | 77 |
| 6/23/2013 | 25 | 2013 | 26 | 78 |
| 6/30/2013 | 23 | 2013 | 27 | 79 |
| 7/7/2013 | 25 | 2013 | 28 | 80 |
| 7/14/2013 | 23 | 2013 | 29 | 81 |
| 7/21/2013 | 25 | 2013 | 30 | 82 |
| 7/28/2013 | 27 | 2013 | 31 | 83 |
| 8/4/2013 | 26 | 2013 | 32 | 84 |
| 8/11/2013 | 24 | 2013 | 33 | 85 |
| 8/18/2013 | 25 | 2013 | 34 | 86 |
| 8/25/2013 | 25 | 2013 | 35 | 87 |
| 9/1/2013 | 27 | 2013 | 36 | 88 |
| 9/8/2013 | 28 | 2013 | 37 | 89 |
| 9/15/2013 | 28 | 2013 | 38 | 90 |
| 9/22/2013 | 29 | 2013 | 39 | 91 |
| 9/29/2013 | 28 | 2013 | 40 | 92 |
| 10/6/2013 | 27 | 2013 | 41 | 93 |
| 10/13/2013 | 27 | 2013 | 42 | 94 |
| 10/20/2013 | 27 | 2013 | 43 | 95 |
| 10/27/2013 | 27 | 2013 | 44 | 96 |
| 11/3/2013 | 27 | 2013 | 45 | 97 |
| 11/10/2013 | 28 | 2013 | 46 | 98 |
| 11/17/2013 | 27 | 2013 | 47 | 99 |
| 11/24/2013 | 24 | 2013 | 48 | 100 |
| 12/1/2013 | 26 | 2013 | 49 | 101 |
| 12/8/2013 | 25 | 2013 | 50 | 102 |
| 12/15/2013 | 23 | 2013 | 51 | 103 |
| 12/22/2013 | 21 | 2013 | 52 | 104 |
| 12/29/2013 | 23 | 2014 | 1 | 105 |
| 1/5/2014 | 27 | 2014 | 2 | 106 |
| 1/12/2014 | 26 | 2014 | 3 | 107 |
| 1/19/2014 | 27 | 2014 | 4 | 108 |
| 1/26/2014 | 28 | 2014 | 5 | 109 |
| 2/2/2014 | 28 | 2014 | 6 | 110 |
| 2/9/2014 | 26 | 2014 | 7 | 111 |
| 2/16/2014 | 27 | 2014 | 8 | 112 |
| 2/23/2014 | 30 | 2014 | 9 | 113 |
| 3/2/2014 | 27 | 2014 | 10 | 114 |
| 3/9/2014 | 27 | 2014 | 11 | 115 |
| 3/16/2014 | 28 | 2014 | 12 | 116 |
| 3/23/2014 | 28 | 2014 | 13 | 117 |
| 3/30/2014 | 28 | 2014 | 14 | 118 |
| 4/6/2014 | 28 | 2014 | 15 | 119 |
| 4/13/2014 | 31 | 2014 | 16 | 120 |
| 4/20/2014 | 29 | 2014 | 17 | 121 |
| 4/27/2014 | 28 | 2014 | 18 | 122 |
| 5/4/2014 | 27 | 2014 | 19 | 123 |
| 5/11/2014 | 26 | 2014 | 20 | 124 |
| 5/18/2014 | 28 | 2014 | 21 | 125 |
| 5/25/2014 | 26 | 2014 | 22 | 126 |
| 6/1/2014 | 35 | 2014 | 23 | 127 |
| 6/8/2014 | 29 | 2014 | 24 | 128 |
| 6/15/2014 | 27 | 2014 | 25 | 129 |
| 6/22/2014 | 27 | 2014 | 26 | 130 |
| 6/29/2014 | 26 | 2014 | 27 | 131 |
| 7/6/2014 | 28 | 2014 | 28 | 132 |
| 7/13/2014 | 27 | 2014 | 29 | 133 |
| 7/20/2014 | 27 | 2014 | 30 | 134 |
| 7/27/2014 | 28 | 2014 | 31 | 135 |
| 8/3/2014 | 27 | 2014 | 32 | 136 |
| 8/10/2014 | 28 | 2014 | 33 | 137 |
| 8/17/2014 | 29 | 2014 | 34 | 138 |
| 8/24/2014 | 29 | 2014 | 35 | 139 |
| 8/31/2014 | 29 | 2014 | 36 | 140 |
| 9/7/2014 | 29 | 2014 | 37 | 141 |
| 9/14/2014 | 34 | 2014 | 38 | 142 |
| 9/21/2014 | 30 | 2014 | 39 | 143 |
| 9/28/2014 | 32 | 2014 | 40 | 144 |
| 10/5/2014 | 30 | 2014 | 41 | 145 |
| 10/12/2014 | 30 | 2014 | 42 | 146 |
| 10/19/2014 | 28 | 2014 | 43 | 147 |
| 10/26/2014 | 30 | 2014 | 44 | 148 |
| 11/2/2014 | 27 | 2014 | 45 | 149 |
| 11/9/2014 | 30 | 2014 | 46 | 150 |
| 11/16/2014 | 29 | 2014 | 47 | 151 |
| 11/23/2014 | 24 | 2014 | 48 | 152 |
| 11/30/2014 | 27 | 2014 | 49 | 153 |
| 12/7/2014 | 26 | 2014 | 50 | 154 |
| 12/14/2014 | 26 | 2014 | 51 | 155 |
| 12/21/2014 | 23 | 2014 | 52 | 156 |
| 12/28/2014 | 27 | 2015 | 1 | 157 |
| 1/4/2015 | 30 | 2015 | 2 | 158 |
| 1/11/2015 | 42 | 2015 | 3 | 159 |
| 1/18/2015 | 33 | 2015 | 4 | 160 |
| 1/25/2015 | 33 | 2015 | 5 | 161 |
| 2/1/2015 | 31 | 2015 | 6 | 162 |
| 2/8/2015 | 32 | 2015 | 7 | 163 |
| 2/15/2015 | 28 | 2015 | 8 | 164 |
| 2/22/2015 | 31 | 2015 | 9 | 165 |
| 3/1/2015 | 29 | 2015 | 10 | 166 |
| 3/8/2015 | 31 | 2015 | 11 | 167 |
| 3/15/2015 | 30 | 2015 | 12 | 168 |
| 3/22/2015 | 31 | 2015 | 13 | 169 |
| 3/29/2015 | 29 | 2015 | 14 | 170 |
| 4/5/2015 | 31 | 2015 | 15 | 171 |
| 4/12/2015 | 30 | 2015 | 16 | 172 |
| 4/19/2015 | 30 | 2015 | 17 | 173 |
| 4/26/2015 | 29 | 2015 | 18 | 174 |
| 5/3/2015 | 31 | 2015 | 19 | 175 |
| 5/10/2015 | 30 | 2015 | 20 | 176 |
| 5/17/2015 | 34 | 2015 | 21 | 177 |
| 5/24/2015 | 30 | 2015 | 22 | 178 |
| 5/31/2015 | 29 | 2015 | 23 | 179 |
| 6/7/2015 | 29 | 2015 | 24 | 180 |
| 6/14/2015 | 29 | 2015 | 25 | 181 |
| 6/21/2015 | 31 | 2015 | 26 | 182 |
| 6/28/2015 | 29 | 2015 | 27 | 183 |
| 7/5/2015 | 33 | 2015 | 28 | 184 |
| 7/12/2015 | 33 | 2015 | 29 | 185 |
| 7/19/2015 | 31 | 2015 | 30 | 186 |
| 7/26/2015 | 31 | 2015 | 31 | 187 |
| 8/2/2015 | 32 | 2015 | 32 | 188 |
| 8/9/2015 | 31 | 2015 | 33 | 189 |
| 8/16/2015 | 30 | 2015 | 34 | 190 |
| 8/23/2015 | 32 | 2015 | 35 | 191 |
| 8/30/2015 | 35 | 2015 | 36 | 192 |
| 9/6/2015 | 34 | 2015 | 37 | 193 |
| 9/13/2015 | 35 | 2015 | 38 | 194 |
| 9/20/2015 | 36 | 2015 | 39 | 195 |
| 9/27/2015 | 37 | 2015 | 40 | 196 |
| 10/4/2015 | 36 | 2015 | 41 | 197 |
| 10/11/2015 | 32 | 2015 | 42 | 198 |
| 10/18/2015 | 34 | 2015 | 43 | 199 |
| 10/25/2015 | 33 | 2015 | 44 | 200 |
| 11/1/2015 | 34 | 2015 | 45 | 201 |
| 11/8/2015 | 33 | 2015 | 46 | 202 |
| 11/15/2015 | 33 | 2015 | 47 | 203 |
| 11/22/2015 | 29 | 2015 | 48 | 204 |
| 11/29/2015 | 32 | 2015 | 49 | 205 |
| 12/6/2015 | 31 | 2015 | 50 | 206 |
| 12/13/2015 | 31 | 2015 | 51 | 207 |
| 12/20/2015 | 28 | 2015 | 52 | 208 |
| 12/27/2015 | 28 | 2015 | 53 | 209 |
| 1/3/2016 | 27 | 2016 | 1 | 210 |
| 1/10/2016 | 29 | 2016 | 2 | 211 |
| 1/17/2016 | 30 | 2016 | 3 | 212 |
| 1/24/2016 | 34 | 2016 | 4 | 213 |
| 1/31/2016 | 33 | 2016 | 5 | 214 |
| 2/7/2016 | 32 | 2016 | 6 | 215 |
| 2/14/2016 | 34 | 2016 | 7 | 216 |
| 2/21/2016 | 38 | 2016 | 8 | 217 |
| 2/28/2016 | 35 | 2016 | 9 | 218 |
| 3/6/2016 | 35 | 2016 | 10 | 219 |
| 3/13/2016 | 34 | 2016 | 11 | 220 |
| 3/20/2016 | 32 | 2016 | 12 | 221 |
| 3/27/2016 | 98 | 2016 | 13 | 222 |
| 4/3/2016 | 42 | 2016 | 14 | 223 |
| 4/10/2016 | 38 | 2016 | 15 | 224 |
| 4/17/2016 | 38 | 2016 | 16 | 225 |
| 4/24/2016 | 35 | 2016 | 17 | 226 |
| 5/1/2016 | 32 | 2016 | 18 | 227 |
| 5/8/2016 | 32 | 2016 | 19 | 228 |
| 5/15/2016 | 32 | 2016 | 20 | 229 |
| 5/22/2016 | 32 | 2016 | 21 | 230 |
| 5/29/2016 | 57 | 2016 | 22 | 231 |
| 6/5/2016 | 100 | 2016 | 23 | 232 |
| 6/12/2016 | 34 | 2016 | 24 | 233 |
| 6/19/2016 | 36 | 2016 | 25 | 234 |
| 6/26/2016 | 39 | 2016 | 26 | 235 |
| 7/3/2016 | 41 | 2016 | 27 | 236 |
| 7/10/2016 | 39 | 2016 | 28 | 237 |
| 7/17/2016 | 35 | 2016 | 29 | 238 |
| 7/24/2016 | 36 | 2016 | 30 | 239 |
| 7/31/2016 | 34 | 2016 | 31 | 240 |
| 8/7/2016 | 31 | 2016 | 32 | 241 |
| 8/14/2016 | 30 | 2016 | 33 | 242 |
| 8/21/2016 | 36 | 2016 | 34 | 243 |
| 8/28/2016 | 36 | 2016 | 35 | 244 |
| 9/4/2016 | 34 | 2016 | 36 | 245 |
| 9/11/2016 | 43 | 2016 | 37 | 246 |
| 9/18/2016 | 45 | 2016 | 38 | 247 |
| 9/25/2016 | 40 | 2016 | 39 | 248 |
| 10/2/2016 | 38 | 2016 | 40 | 249 |
| 10/9/2016 | 36 | 2016 | 41 | 250 |
| 10/16/2016 | 36 | 2016 | 42 | 251 |
| 10/23/2016 | 35 | 2016 | 43 | 252 |
| 10/30/2016 | 36 | 2016 | 44 | 253 |
| 11/6/2016 | 32 | 2016 | 45 | 254 |
| 11/13/2016 | 36 | 2016 | 46 | 255 |
| 11/20/2016 | 29 | 2016 | 47 | 256 |
| 11/27/2016 | 35 | 2016 | 48 | 257 |
| 12/4/2016 | 34 | 2016 | 49 | 258 |
| 12/11/2016 | 32 | 2016 | 50 | 259 |
| 12/18/2016 | 32 | 2016 | 51 | 260 |
| 12/25/2016 | 37 | 2016 | 52 | 261 |

**Figure S1. Classical Decomposition of the Sepsis Relative Search Volume Time Series**

The sepsis relative search volume time series was subjected to outlier replacement via linear interpolation, a third order simple moving average was taken, and the resulting time series was subjected to classical decomposition.

**Figure S2. Linear Model for the United States Sepsis (Topic) Relative Search Volume Time Series**

The United States sepsis relative search volume time series was subjected to adjustment for outliers (red tracing) and then linear regression (blue tracing; intercept = 22.1, slope = 0.048, R^2^ = 0.72, p = <0.0001).
